# TGF‐β regulates the release of breast cancer cell‐derived extracellular vesicles and the sorting of their protein cargo by downregulating RAB27B expression

**DOI:** 10.1002/jev2.70026

**Published:** 2024-12-26

**Authors:** Chao Li, Agustin Enciso‐Martinez, Roman I. Koning, Mona Shahsavari, Peter ten Dijke

**Affiliations:** ^1^ Oncode Institute and Department of Cell and Chemical Biology Leiden University Medical Center Leiden Netherlands; ^2^ Biomedical Engineering & Physics; Laboratory of Experimental Clinical Chemistry, Laboratory Specialized Diagnostics & Research, Department of Laboratory Medicine Amsterdam University Medical Center, Meibergdreef 9 Amsterdam Netherlands; ^3^ Electron Microscopy Facility, Department of Cell and Chemical Biology Leiden University Medical Center Leiden Netherlands; ^4^ Amsterdam Cardiovascular Sciences Atherosclerosis and Ischemic Syndromes Amsterdam Netherlands; ^5^ Cancer Center Amsterdam, Imaging and Biomarkers Amsterdam Netherlands

**Keywords:** CD8^+^ T cells, extracellular vesicles, multivesicular bodies, RAB27B, release, TGF‐β

## Abstract

Extracellular vesicles (EVs) are important mediators of intercellular communication in the tumour microenvironment. The cytokine transforming growth factor‐β (TGF‐β) facilitates cancer progression via EVs secreted by cancer cells, which act on recipient cells in the tumour microenvironment. However, the mechanisms of how TGF‐β affects cancer cell EV release and composition are incompletely understood. Here, we systematically investigate the effects of TGF‐β on the release and protein composition of EVs from breast cancer cells. TGF‐β suppresses the transcription of *RAB27B* mediated by SMAD3 and thereby hampers EV release. Using click chemistry and quantitative proteomics, we found that TGF‐β increases the quantity of protein cargo and changes the composition of EVs by downregulating RAB27B expression. The recomposed EVs, induced by TGF‐β or RAB27B depletion, inhibit CD8^+^ T cell‐mediated breast cancer killing. Our findings reveal the critical roles of TGF‐β and RAB27B in cancer development by regulating EV release and composition and thus provide potential targets to improve cancer immunotherapy.

## 1 INTRODUCTION

Extracellular vesicles (EVs) are cell‐released lipid bilayer‐enclosed particles. EVs are heterogeneous in biochemical composition, function, and biogenesis. They can be derived from the intraluminal vesicles (ILVs) within multivesicular bodies (MVBs) as exosomes, or from the direct budding of the plasma membrane as ectosomes (Cocucci & Meldolesi, 2015). EVs are thought to be important mediators in intercellular communication by either making functional membrane contacts (Rausch et al., 2023; Shelke et al., 2019) or by transferring bioactive cargo to recipient cells, thereby regulating cellular homeostasis, and contributing to both physiological and pathological processes (van Niel et al., 2018).

In the tumour microenvironment, EV‐mediated signalling between cancer cells and stromal cells facilitates tumour growth, invasion and immune evasion (Kalluri & McAndrews, 2023; Xu et al., 2018). Transforming growth factor‐β (TGF‐β) is a secreted cytokine with a pivotal role in tumour development and immune escape (Colak & Ten Dijke, 2017). TGF‐β exerts its effects via cell surface TGF‐β type I and type II serine/threonine kinase receptors (i.e., TβRI and TβRII) and intracellular SMAD transcription factors (Heldin & Moustakas, 2016). Growing evidence shows that there is an interplay between TGF‐β and EVs (Hosseini et al., 2023; Shelke et al., 2019; Teixeira et al., 2023; Xie et al., 2022). Specially, TGF‐β1 (Shelke et al., 2019; Teixeira et al., 2023; F. Zhang et al., 2023) and one of its cell surface receptors, that is, TβRII receptor (Xie et al., 2022), have been identified as cargo of cancer cell‐derived EVs. The transfer of these cargo into neighbouring cancer and stromal cells can promote TGF‐β signalling and thereby contribute to cancer metastasis and immune evasion (Kobayashi et al., 2022; Teixeira et al., 2023). However, whether TGF‐β has effects on EV release, composition and function remains largely unknown.

The release of EVs consists of a series of well‐organized membrane dynamic processes. The quantitative analysis of EV release is challenging as it requires (1) quantitative EV isolation methods, preferably with high recovery and purity, and (2) sensitive and accurate quantification techniques, for example, for EV concentration measurements. Our study quantified EV release using microfluidic resistive pulse sensing (MRPS) and nanoparticle tracking analysis (NTA) (Arab et al., 2021; Fraikin et al., 2011; Gandham et al., 2020). We also used the pHluorin‐CD63 reporter (Bebelman et al., 2020), an EV isolation and detection independent method, to detect the dynamic multivesicular body‐plasma membrane (MVB‐PM) fusion events and the secretion of exosomes.

The RAB family proteins of small GTPases, in particular RAB27A and RAB27B (Matsui et al., 2022; Ostrowski et al., 2010; Takahashi et al., 2019), play an important role in vesicular trafficking, including MVB transport and docking and in EV secretion (Matsui et al., 2022; Ostrowski et al., 2010; Takahashi et al., 2019). Knocking down these two genes has been widely used to inhibit the release of EVs (Sung et al., 2020; Teixeira et al., 2023). In our study, we found that TGF‐β signalling inhibits the mRNA and protein expression of RAB27A and RAB27B in a SMAD3‐dependent manner, leading to reduced EV release in breast cancer cells.

Although RAB27B plays a role in regulating MVB trafficking and EV secretion, it is unknown whether RAB27B affects the composition of EV cargo. Here, we studied whether RAB27B affects the EV cargo by using click chemistry to label newly synthesized proteins in EVs, and MRPS‐based quantitative proteomics analysis, and we demonstrate that TGF‐β upregulates the quantity of protein cargo and alters the protein composition in EVs in breast cancer cells primarily mediated by the downregulation of RAB27B expression. Furthermore, we isolated MVBs to determine whether TGF‐β and RAB27B affect the protein cargo loading into exosomes. We found the quantity of protein significantly increased, indicating that both TGF‐β and RAB27B regulate cargo loading processes for exosomes. The breast cancer cell‐derived EVs with such recomposed cargo impede the cancer cell killing of activated CD8^+^ T cells. Taken together, we identified that TGF‐β in breast cancer cells reduces the release of EVs, but upregulates the quantity of protein loaded into EVs, particularly exosomes, by downregulating the expression of RAB27B. The TGF‐β‐induced recomposed EVs lead to immunosuppression of CD8^+^ T cells.

## 2 MATERIALS AND METHODS

### 2.1 Cell lines

HEK293T (CRL‐1573), MDA‐MB‐231 (CRM‐HTB‐26), MDA‐MB‐436 (HTB‐130), BT‐549 (HTB‐122), MCF‐7 (HTB‐22), BT‐474 (HTB‐20), and HeLa (CCL‐2) cells were purchased from the American Type Culture Collection (ATCC). SUM149 cells were obtained from Dr. Sylvia Le Dévédec (Leiden Academic Center for Drug Research, Leiden, the Netherlands). BT‐474 cells were cultured in RPMI 1640 medium (Thermo Fisher Scientific, 21875034), and other cell lines were cultured in Dulbecco's modified Eagle medium (DMEM, Thermo Fisher Scientific, 41966029). The RPMI 1640 and DMEM medium were supplemented with 10% (v/v) foetal bovine serum (FBS, S1810, BioWest), 100 U/mL penicillin and 100 µg/mL streptomycin (Thermo Fisher Scientific, 15140163). All cell lines were maintained in a 37°C and 5% CO_2_ humidified incubator, tested routinely for mycoplasma contamination, and checked for authenticity by short tandem repeat profiling.

### 2.2 T cell isolation, activation, and culture

CD8^+^ T cells were cultured in Iscove's Modified Dulbecco's Medium (IMDM, Thermo Fisher Scientific, 21980032) supplemented with 8% (v/v) heat‐inactivated human serum (PAN‐Biotech, P40‐2701), 4 mM L‐glutamine (Thermo Fisher Scientific, A2916801), 50 U/mL penicillin and 50 µg/mL streptomycin (Thermo Fisher Scientific, 15140163).

CD8^+^ T cells were isolated from the peripheral blood mononuclear cells (PBMCs) of healthy blood donors in accordance with the Declaration of Helsinki and the Dutch rules with respect to the use of human materials from volunteer donors. PBMCs were isolated by density gradient centrifugation using Histopaque‐1077 (Sigma, 10771) according to the manufacturer's instructions. Briefly, fresh peripheral blood was collected in sodium heparin tubes and layered on the top of Histopaque‐1077 solution. After centrifugation at 400 × *g* for 30 min at room temperature, the PBMCs in the opaque interface were transferred to a new tube, and then washed with phosphate buffered saline (PBS) for three times. PBMCs were spun down at 250 × *g* for 10 min after each washing step. CD8^+^ T cells were further purified by negative isolation with depletion Dynabeads and antibody mix targeting other cell types in PBMCs (Thermo Fisher Scientific, 11348D) according to the manufacturer's instructions. After isolation, CD8^+^ T cells were resuspended in culture medium with a 1 × 10^6^ cells/mL concentration.

T cells were activated using Dynabeads Human T‐Activator CD3/CD28 beads (Thermo Fisher Scientific, 11131D). The pre‐washed Dynabeads were added to the CD8^+^ T cells to obtain a bead‐to‐cell ratio of 1:1 for 3 days. After activation, the beads were removed with a magnet (Thermo Fisher Scientific, 12321D) and T cells were kept for culturing with 5 ng/mL recombinant human IL‐2 (PeproTech, 200–02).

### 2.3 Plasmids

The cDNA fragments encoding full open reading frames of human *RAB27B* and *SMAD3* were amplified by PCR from the cDNA of MDA‐MB‐231 cells. The *RAB27B* was cloned into pLV‐cytomegalovirus (CMV)‐intra ribosomal entry site (IRES)‐puromycin (PURO) with a C‐terminal Flag‐tag using *Spe*I restriction sites, and the *SMAD3* was cloned into pLV‐CMV‐IRES‐neomycin (NEO) using *Mlu*I/*Xho*I restriction sites. *RAB27A* and *RAB27B* promoter fragments were amplified by PCR from MDA‐MB‐231 genomic DNA and cloned into the pGL4‐luc using *Xho*I/*Hind*III restriction sites. The short hairpin RNA (shRNA) constructs for the knockdown of *RAB27A, RAB27B*, and *SMAD3* were obtained from Mission shRNA library of Sigma‐Aldrich. TRCN0000005296 and TRCN0000005297 were used for *RAB27A* knockdown, targeting the CDS region of *RAB27A*. TRCN0000293978 and TRCN0000294016 were used for *RAB27B* knockdown, targeting the CDS and 3′ UTR of *RAB27B*, respectively. TRCN0000020009 and TRCN00000200010 were used for *SMAD3* knockdown, targeting the CDS region of *SMAD3*. The pLenti‐pHluorin M153R‐CD63 and pLenti‐pHluorin M153R‐CD63‐mScarlet were purchased from Addgene (172117 and 172118). All modified plasmids were verified by sanger sequencing.

### 2.4 Lentiviral packaging and generation of stable cell lines

A third‐generation lentiviral packaging system (pRSV‐Rev, pMDLg/pRRE, and pCMV‐VSV‐G) was used for lentiviral production. Lentiviral envelope, packaging, and transfer plasmids were transfected into HEK293T using polyethylenimine (PEI, Polysciences, 23966) for lentiviral production. The medium was changed at 16 h post‐transfection. Cell supernatants were collected at 48 –72 h post‐transfection and filtered through 0.45‐µm polyvinylidene fluoride (PVDF) membrane filters (Millipore, SLHVR33RB). To generate stable cell lines, cells were seeded in 12‐well plates to achieve 60%–70% confluency the following day and infected by lentiviral supernatants supplemented with the same volume of fresh medium. To the cells with lentivirus, 8 ng/mL Polybrene (Sigma‐Aldrich, 107689) was added. After 24 h of infection, cells were expanded and cultured with the corresponding antibiotics for selection.

### 2.5 Preparation of conditioned medium and EV isolation

EV‐depleted FBS was prepared using normal FBS filtered by 100‐kDa Amicon ultra‐15 centrifugal filters (Millipore, UFC910024) at 3200 × *g* in a swinging‐bucket rotor (Kornilov et al., 2018).

Cells were seeded in 15‐cm culture dishes with completed medium, and recombinant human TGF‐β3 (2.5 ng/mL) or ligand buffer (vehicle control) was added to the cells for the pretreatment on the following day for 24 h. After pretreatment, the medium was discarded, and cells were washed with 1 × PBS. Serum‐free medium was added to the MDA‐MB‐231, BT‐549, and MCF‐7 cells, and medium supplemented with EV‐depleted FBS was added to the SUM149, BT‐474 and HeLa cells, with TGF‐β3 (2.5 ng/mL) or ligand buffer. After 48 h, conditioned medium of each condition was harvested by pipetting from the dishes. At the time point of collection, the cells reached 95% confluency. The cell number and viability were measured after collecting the conditioned medium using TC20 automated cell counter (Bio‐Rad, 1450102) and staining with trypan blue (Bio‐Rad, 1450013), and the cell viability was above 95%.

For EV isolation for most of experiments in this study, the conditioned medium was centrifuged at 300 × *g* for 5 min at 4°C to remove cells. The supernatant was centrifuged at 2000 × *g* for 15 min, followed by 3200 × *g* for 15 min at 4°C to remove cell debris. The supernatant was concentrated using 100‐kDa Amicon ultra‐15 centrifugal filters at 3200 × *g* to a volume of 300 µL. All centrifugation steps were conducted using a swinging‐bucket rotor. The concentrated medium was transferred to 1.5‐mL protein low‐binding tubes (Eppendorf, 0030108116). The filters were washed by adding an additional 200 µL serum‐free medium. Two volumes of medium were mixed to achieve a final volume of 500 µL for size exclusion chromatography (SEC). The EVs were further purified by SEC using 35‐nm qEV size‐exclusion columns (IZON, ICO‐35) and automatic fraction collector‐V2 (IZON, AFC‐V2) according to the manufacturer's instructions. The SEC columns and samples were recovered to room temperature first. The buffer volume was 2.5 mL, and the purified collection volume was 2 mL in one fraction on AFC‐V2. The samples were loaded on the loading frit of SEC columns, and the buffer volume was collected immediately. The PBS filtered through 0.22‐µm filters was topped up when all the samples entered the frit. After the buffer volume reached 2.5 mL, the 2‐mL EV samples were collected into 2‐mL protein low‐binding tubes (Eppendorf, 0030108132). The purified EV samples were aliquoted and snap‐frozen in liquid nitrogen if the samples were not used immediately. The EV samples were kept at −80°C after snap freezing.

For the EV isolation for cryo‐EM, the supernatant of the conditioned medium after differential centrifugation was concentrated by tangential flow filtration (TFF)‐based devices (Hansabiomed, HBM‐TFF‐EVs‐S) with hollow fibre filters with 50 ± 10 nm pore size at a flow rate of 10 mL/min. The volume of the concentrated medium after TFF was 500 µL. The samples were then loaded on SEC columns for further EV purification as described before. The 2‐mL purified EVs in PBS after SEC were then concentrated using EV spinners (Hansabiomed, HBM‐EVS100‐24) at 2000 × *g* in a swinging‐bucket rotor to reach a final volume of 100–150 µL. The samples were then aliquoted in 1.5‐mL protein low‐binding tubes, snap‐frozen in liquid nitrogen and kept at −80°C.

The abbreviations con‐EVs and TGF‐β‐EVs refer to EVs derived from vehicle buffer‐treated cells and TGF‐β‐treated cells, respectively. The abbreviation shRAB27B‐EVs refers to EVs derived from shRNA‐mediated *RAB27B* knockdown cells. In the experiments involving shRAB27B‐EVs, con‐EVs and TGF‐β‐EVs were derived from control lentivirus‐infected cells.

### 2.6 Nanoparticle tracking analysis (NTA)

The NTA was conducted using NanoSight NS300 with a 488‐nm laser and NTA software v3.2. The EVs samples were diluted in PBS, which was filtered through 0.22‐µm filters, to obtain a range of concentrations that allow detection of 10–100 particles per frame during measurement. The temperature was set at 21°C, and the camera level was set at 13 to have most of the small particles appearing in the sight with minimum background noise. The samples were loaded into chamber using syringes and captured for 60 s for three repeats, and the images were captured at a rate of 25 frames/s. The recorded videos were then analysed with a detection threshold of 5 to estimate the concentration and size distribution of EVs, and the settings were kept consistent for all samples.

### 2.7 Microfluidic resistive pulse sensing (MRPS)

The MRPS assay was conducted using nCS1 (v0, Spectradyne) with C‐400 cartridges (specified size range: 65–400 nm). Poloxamer‐188 (Alfa Aesar, J66087) with a concentration of 1% (w/v) in Dulbecco's Phosphate‐Buffered Saline (DPBS) (Corning, 21‐031‐CVR) was filtered through 0.05‐µm Isopore filters (Whatman) before measurements. EV samples were diluted in DPBS with 0.05% (w/v) poloxamer‐188 (Shahsavari et al., 2024), and 6 µL of samples were loaded into cartridges for detection. Default settings were used for the data acquisition. The results were further processed using nCS1 Viewer software (v2.5.0.297, Spectradyne). The false positive events were excluded by setting the peak filters as follows: transit time < 60 µs and < 100 µs based on Mold‐ID of the cartridge; symmetry: 0.2–0.4; signal to noise > 10; diameter > 65 nm. A bin size of 10 nm was chosen to show the measured particle size distributions, and the reported concentrations included particles within a size range of 70 to 400 nm.

### 2.8 EV labelling and uptake assay

EVs were labelled with PKH67 dye (Sigma‐Aldrich, MINI67) according to the manufacturer's instructions with slight modifications. The purified EV samples after SEC were spined down using ultracentrifugation at 120,000 × *g* for 90 min at 4°C, and the EVs were resuspended with Diluent C. The dye solution was prepared by diluting the PKH67 dye with the same volume of Diluent C to the working concentration of 4 µM. The EV suspension was added to the dye solution and mixed by gentle pipetting, and the mixture was incubated at room temperature for 5 min. The dye was quenched with 1% BSA in filtered PBS and incubated for 1 min. As described before, the labelled EVs were purified with SEC and concentrated with EV‐spinners. The labelled EVs were incubated with target cells for 4 h before detection.

### 2.9 Immunoblotting (western blot)

For protein analysis of EV samples, the proteins were concentrated by trichloroacetic acid (TCA) precipitation as described (X. Zhang et al., 2020). First, sodium deoxycholate was added to the EV samples with a concentration of 2 mg/mL. The ice‐cold 100% (w/v) TCA (Sigma‐Aldrich, 91230) was then added to samples with a concentration of 20% and mixed immediately. The samples were incubated for 30 min at 4°C, and centrifuged at 16,000 × *g* for 10 min at 4°C. The supernatant was discarded, and the pellets were washed twice with 1 mL of pre‐cooled acetone and centrifuged as above. The protein pellets were air dried, resuspended in lysis buffer (10% [w/v] glycerol, 2% [w/v] SDS, and 60 mM Tris‐HCl [pH 6.8]) and heated at 95°C for 5 min. For protein analysis of cell samples, the cells were washed with PBS and lysed with the lysis buffer described above, and the cell lysates were heated at 95°C for 5 min. The protein concentrations were determined using the bicinchoninic acid (BCA) protein assay (Thermo Fisher Scientific, 23227), according to the manufacturer's instructions.

Proteins were separated by SDS‐polyacrylamide gel electrophoresis (PAGE) followed by transferring to 0.45‐µm PVDF membranes (Millipore, IPVH00010). Membranes were blocked with 5% (w/v) non‐fat milk in TBST buffer (20 mM Tris‐HCl [pH 7.6], 150 mM NaCl, and 0.1% [v/v] Tween 20) for 1 h at room temperature. The membranes were incubated with primary antibodies diluted with 3% BSA in TBST buffer at 4°C overnight and secondary antibodies diluted with 5% (w/v) non‐fat milk in TBST buffer at room temperature for 1 h. The membranes were washed four times with TBST buffer for 5 min after each incubation. The horseradish peroxidase (HRP) signals were detected with enhanced chemiluminescent (ECL) substrate (Bio‐Rad, 170–5061) and ultra‐sensitive ECL substrate (Thermo Fisher Scientific, 34095) using a Bio‐Rad ChemiDoc imaging system. The results were further processed using Image Lab software. The antibodies used for western blot are listed in Table S1.

### 2.10 RNA extraction and quantitative real‐time PCR

Total RNA was isolated from cells using NucleoSpin RNA kits (Macherey Nagel, 740955), according to the manufacturer's protocol. The RNA concentrations were measured using NanoDrop One (Thermo Fisher Scientific), and 1 µg RNA per sample was used for cDNA synthesis using RevertAid H Minus reverse transcription kit (Thermo Fisher Scientific, K1632). Quantitative PCR was performed using the GoTaq qPCR Master mix (Promega, A600X) on the CFX Connect Real‐Time PCR detection system (Bio‐Rad), and the data was acquired using Bio‐Rad CFX Manager v3.1 software. The housekeeping gene glyceraldehyde 3‐phosphate dehydrogenase (GAPDH) was used to calculate 2^−ΔΔCt^. The primer sequences used for RT‐qPCR are listed in Table S2.

### 2.11 Luciferase reporter assay

HEK293T or HeLa cells were seeded in 24‐well plates to achieve 70% confluency the following day. One hundred fifty nanogram pGL4‐luc containing *RAB27A* or *RAB27B* promoter regions and 100 ng β‐galactosidase expression construct were transfected into cells with PEI. After 16 h, the medium was refreshed with TGF‐β3 (2.5 ng/mL) or same volume of ligand buffer for 24 h. Luciferase activities in the cell lysates were measured with the luciferase assay substrates (Promega, E151A and E152A) and a luminometer (PerkinElmer), and values were normalized for differences in transfection efficiency by measuring β‐galactosidase activity from the same cell extracts.

### 2.12 Flow cytometry

T cells were treated with EVs or PBS control for 2 days. After 2 days, T cells were pretreated with cell stimulation cocktail (Thermo Fisher Scientific, 00‐4970‐93) and protein transport inhibitor cocktail (Thermo Fisher Scientific, 00‐4980‐03) for 6 h. T cells were washed with PBS and stained with Zombie Violet dye (BioLegend, 423113) in PBS at 4°C for 30 min. For surface staining, cells were washed with FACS buffer (PBS supplemented with 0.1% [w/v] NaN_3_ and 5% [v/v] FBS) once, and then stained with fluorophore‐conjugated antibodies in FACS buffer at 4°C for 30 min in dark. For intracellular staining, cells were washed after surface staining and fixed by 4% formaldehyde (Thermo Fisher Scientific, 20908) for 20 min at room temperature. After fixation, cells were washed once with PBS and permeabilized with permeabilization buffer (PBS supplemented with 0.1% [w/v] saponin and 0.5% [w/v] BSA) for 10 min at room temperature. The fluorophore‐conjugated antibodies were added to the cells for intracellular staining at 4°C for 30 min. The cells were washed three times with permeabilization buffer after staining. Samples were analysed using Cytek Aurora flow cytometers, and the results were further processed using FlowJo v10.8. FlowAI or FlowClean was used to control the quality of results. A complete list of flow cytometry antibodies can be found in Table S3.

### 2.13 Enzyme‐linked immunosorbent assay (ELISA) assay

T cells were treated with EVs or a PBS control for 2 days. After 2 days, the supernatants of T cells were collected for the detection of interferon‐gamma (IFN‐γ) and tumor necrosis factor‐alpha (TNF‐α). The Elisa experiments were performed using Human IFN‐γ (ALP) ELISA kits (MABTECH, 3420–1A‐6) and Human TNF‐α (ALP) ELISA kits (MABTECH, 3512‐1A‐6) according to the manufacturer's instructions. The signals were measured at 405 nm using a plate reader (PerkinElmer).

### 2.14 Killing assay

BT‐474 mCherry cells were seeded in 96‐well plates with a density of 5000 cells/well. On the following day, the activated CD8^+^ T cells were seeded to the plates with a effector:target (E:T) ratio of 4:1, and the anti‐human epidermal growth factor receptor (HER)2 × anti‐CD3 epsilon (CD3E) bispecific antibody (100 ng/mL, Absolute Antibody, bAb0183) and corresponding treatments were added with CD8^+^ T cells to the plates. The cells were cultured in the Incucyte S3 (Sartorius) and recorded for 2–3 days.

### 2.15 Immunofluorescence assay

Cells were seeded on the coverslips (1.5 H thickness, Roth, YX03.1) in 24‐well plates for the immunofluorescent staining. After treatments, cells were fixed with 4% (v/v) formaldehyde (Thermo Fisher Scientific, 20908) for 15 min at room temperature. Cells were washed three times with PBS and permeabilized with 0.2% (v/v) Triton X‐100 for 10 min at room temperature, followed by three washes with PBS. Cells were blocked with 3% (w/v) BSA in PBS for 30 min at room temperature, and incubated with primary antibodies overnight at 4°C. After washing three times with PBS, cells were incubated with fluorophore‐conjugated secondary antibodies for 1 h at room temperature. All antibody dilutions were centrifuged at 16,000 × *g* for 5 min at 4°C to remove antibody aggregates. The slides were washed and mounted with antifade mounting medium with 4′, 6‐diamidino‐2‐phenylindole (DAPI) (Vector Laboratories, H‐1500). Samples were imaged using Zeiss LSM 900, and the Airyscan detector was used for super‐resolution imaging. Images were processed using ZEN v3.6 and Fiji software.

### 2.16 Total internal reflection fluorescence (TIRF) imaging

Cells were cultured in glass‐bottom dishes (Greiner Bio‐One, 627870) and maintained in a 37°C incubator with 5% CO_2_, and treatments were added to the cells for 2 days. TIRF was performed using Andor Dragonfly 500 equipped with a 37°C chamber, a Zyla scientific Complementary Metal–Oxide–Semiconductor (sCMOS) camera and a HC PL APO 100x/1,47 OIL CORR TIRF lens. Highly inclined and laminated optical sheet (HILO) illumination was used for TIRF mode and the value was set at 1° to obtain the optimal signal‐to‐noise signal. The live imaging was conducted at 5 Hz. Results of live imaging were analysed with Imaris v9.5 and Fiji software.

### 2.17 Electron microscopy

Quantifoil 2/2 grids were glow discharged in air at 0.2 mbar, 25 mA, and 30 s using a Pelco Easyglow. A 3‐µL sample was added to the grid and blotted away for 3 s using filter paper (Whatman no.4) at 85%–95% humidity and room temperature using a Leica EM GP. The grid was subsequently plunged into liquid ethane/propane kept at −196°C. Grids were transferred to the Talos Arctica (Thermo Fisher Scientific) operated by EPU software in multi‐grid mode. Images were recorded using a K3 direct electron detector (Gatan) in counting mode and zero‐loss peak (ZLP) imaging in movie mode at a magnification of 15,000x (corresponding to a pixel size of 0.55 nm at specimen level), a defocus of −5 micron, and a total dose of 35 e/A^2^. Movies were aligned using MotionCor2 (Zheng et al., 2017) and converted to tiff using EMAN2 (Tang et al., 2007).

### 2.18 Mass spectrometry (MS)

For label‐free proteomic analysis, three biological replicates were prepared for each group. EV proteins were isolated using TCA protein precipitation as described before, and the protein pellet of each sample was resuspended with 40 µL ammonium bicarbonate (50 mM). Protein samples were quantified and 1.25 µg of each sample was used for in‐solution digestion. The trypsin was added to the protein samples with a ratio of 1:20 (w/w), and the mixtures were incubated at 37°C overnight. The peptide fractions were freeze‐dried and reconstituted with mobile phase A (2% acetonitrile and 0.1% formic acid). After centrifugation at 20,000 × *g*  for 10 min, the supernatant was loaded for the liquid chromatography mass spectrometry (LC‐MS)/MS analysis using an UltiMate 3000 ultra‐high performance liquid chromatography (UHPLC) (Thermo Fisher Scientific) coupled to a timsTOF Pro mass spectrometer (Bruker). The peptide samples were loaded on a trap column connected to the self‐packed C18 column (32 cm column length, 150 µm inner diameter, 1.8 µm column material particle size), and separated using a linear gradient of mobile phase B buffer (84% acetonitrile and 0.1% formic acid) at a flow rate of 500 nL/min. The peptides separated by liquid phase chromatography were ionized by a nano electrospray ionization (nanoESI) source and then passed to a timsTOF Pro mass spectrometer for data dependent acquisition (DDA) mode detection. The ion source voltage was set to 1.6 kV. The MS survey scans were performed with a range of 100–1700 m/z at a resolution of 50,000. MS/MS data were acquired in positive ionization mode, and the first 10 precursor ions with a peak intensity exceeding 10,000 and a peak intensity above 2500 can be detected. The MS/MS cumulative scan time was set to 100 ms, and collision‐induced dissociation (CID) mode was used for the ion fragmentation. The dynamic exclusion time was set to 30 s.

### 2.19 Proteomics analysis

The raw MS files were processed using MaxQuant v2.1.3.0 and searched against the human reference proteome database using the Andromeda engine. The parameters included a minimal peptide length of seven amino acids, specificity for trypsin, and a maximum of two missed cleavages. Carbamidomethyl (C) was set as a fixed modification, and oxidation (M), acetyl (Protein N‐term) and deamidated (NQ) were set as variable modifications. Match between runs was used with a matching time window of 0.7 min and an alignment time window of 20 min. The false discovery rate (FDR) was 1% for peptides and protein levels.

The label‐free quantification intensities were further analysed using Perseus v2.0.11 software. Data were filtered to remove reverse peptides and proteins identified only by site, and proteins identified with more than one unique peptide were used for analysis. The intensity values were log2‐transformed and the proteins that have at least two valid values in at least one group remained. Missing values were imputed. Statistic differences were assessed by two‐sided student's *t*‐test or one‐way analysis of variance (ANOVA). Proteins with an absolute log2 fold change (Log_2_FC) > 1 and *p*‐value < 0.05 between two groups were recognized as differentially expressed proteins (DEPs).

### 2.20 Bioinformatic analysis

Gene ontology (GO) and reactome pathway enrichment analyses were performed on their corresponding website (https://geneontology.org/, https://reactome.org/) and KOBAS (http://bioinfo.org/kobas). The enriched pathways were visualized using the R packages.

### 2.21 Click‐it chemistry

Cells were washed with PBS, and the medium was changed to methionine‐free DMEM medium (Thermo Fisher Scientific, 21013024) supplemented with 0.2 mM L‐cystine dihydrochloride, 4 mM L‐glutamine, 1 mM sodium pyruvate, 100 U/mL penicillin, and 100 µg/mL streptomycin. Cells were incubated with methionine‐free medium for 1 h to deplete methionine reserves, L‐azidohomoalaine (AHA, Thermo Fisher Scientific, C10102) or L‐methionine (Thermo Fisher Scientific, J61904.22) was added to the medium with a concentration of 50 µM. For the immunofluorescence assay, the cells were treated with AHA or methionine for 4, 8 and 12 h. The cells were fixed and permeabilized as described above and incubated with 5 µM Alexa Fluor 488‐DBCO (Jena Bioscience, CLK‐1278‐1) for 30 min at room temperature. Samples were imaged using Zeiss LSM 900. For EV preparation, TGF‐β3 (2.5 ng/mL) or ligand buffer control was added to the cells with AHA or methionine for 8 h. The cells were washed with PBS and cultured using serum‐free medium with TGF‐β3 (2.5 ng/mL) or ligand buffer control for 48 h. The conditioned medium was collected for the EV isolation. The cell and EV samples were lysed with lysis buffer (1% SDS, 50 mM Tris‐HCl [pH 8.0]) and incubated with 40 µM DBCO‐PEG_4_‐Biotin (Jena Bioscience, CLK‐A105P4‐10) for 1 h at 37°C. Newly synthesized proteins were visualized by performing the western blot assay using the streptavidin‐HRP antibody.

### 2.22 Late endosome isolation

Late endosomes were isolated according to previously reported methods (Clement et al., 2021). Briefly, cells were washed with PBS and centrifuged at 300 × *g* for 3 min. The cells were resuspended in 1 mL PBS containing 2.5 M sucrose, 20 mM 4‐(2‐hydroxyethyl)‐1‐piperazineethanesulfonic acid (HEPES) (pH 7.4), 1 mM EDTA, and protease inhibitor (Roche, 11836145001) and homogenized in Dounce homogenizers on ice for 10 min. After centrifugation at 150 × *g* for 10 min at 4°C, the supernatant was collected and the pellet was resuspended and homogenized with the same procedures. Two parts of supernatants were combined and loaded on 9 mL of 27% Percoll (Sigma‐Aldrich, P1644) laid above 1 mL of 2.5 M sucrose in 20 mM HEPES (pH 7.4), and the samples were centrifuged at 34,000 × *g* for 1 h at 4°C in a SW40 Ti rotor. The fractions enriched with late and early endosomes were collected and loaded on 9 mL of 10% Percoll laid above 1 mL of 2.5 M sucrose in 20 mM HEPES (pH 7.4), and the samples were centrifuged at 34,000 × *g* for 1 h at 4°C in a SW40 Ti rotor. The fractions enriched with late endosomes were collected and washed with PBS containing 0.25 M sucrose and 20 mM HEPES (pH7.4), and centrifuged at 3000 × *g* for 10 min at 4°C.

### 2.23 Statistics

All statistical analyses were performed using Graphpad Prism 10. Unless otherwise indicated, the results are shown as mean ± standard deviation (SD). Two‐sided statistical tests were performed in all statistical analyses. Differences were considered statistically significant at *p* < 0.05.

## 3 RESULTS

### 3.1 TGF‐β affects the EV release and morphology in breast cancer cells

**FIGURE 1:**
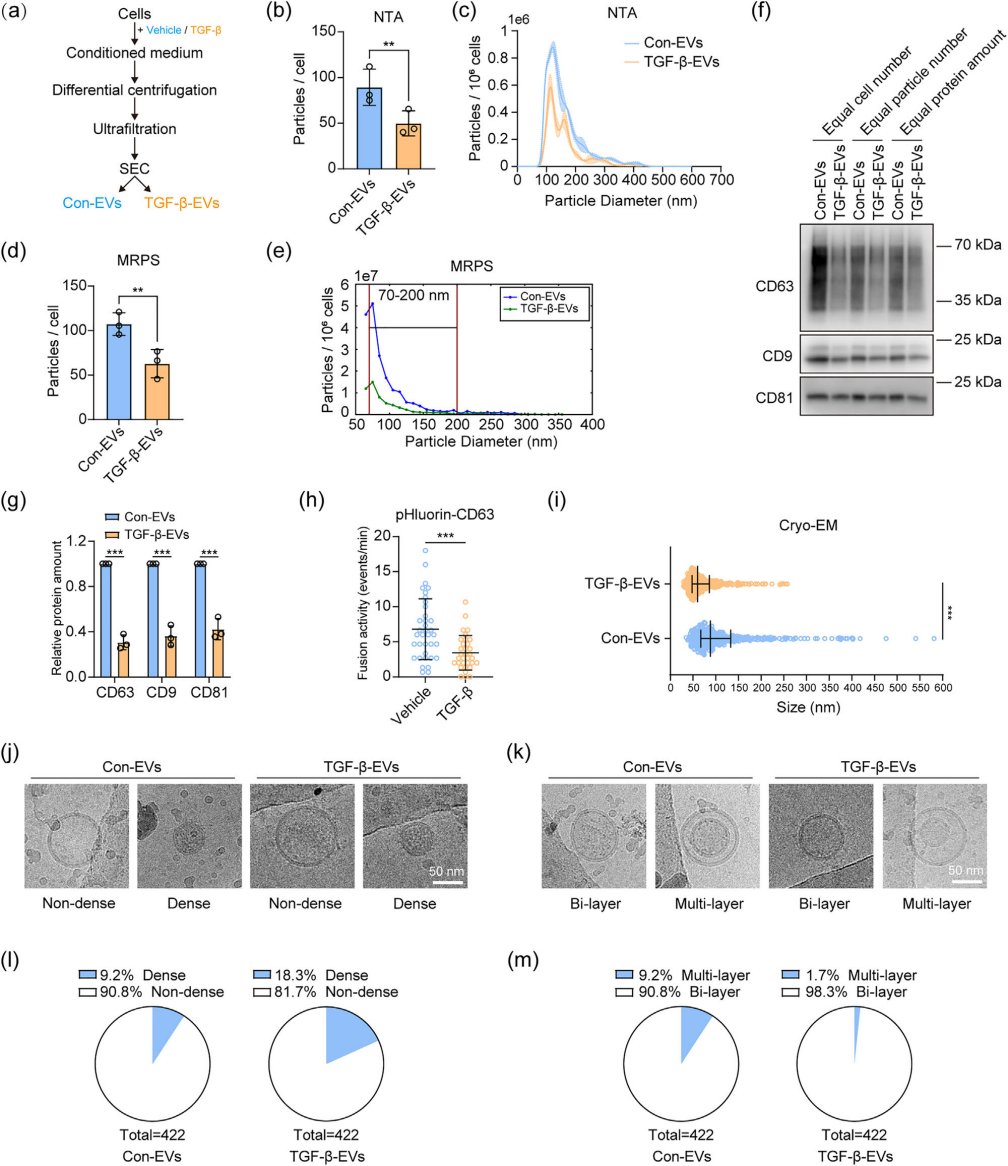
TGF‐β affects the EV release and morphology. (a) Schematic of the EV isolation workflow. Cells were treated with vehicle buffer or TGF‐β. Con‐EVs and TGF‐β‐EVs were isolated from corresponding treated cells. (b, c) EV release and representative size distribution of con‐EVs and TGF‐β‐EVs characterized by nanoparticle tracking analysis (NTA). The total EV numbers were normalized to total EV‐releasing cells. Means ± SD, *n* = 3 independent experiments, paired student's *t*‐test. (d, e) EV release and representative size distribution of con‐EVs and TGF‐β‐EVs characterized by microfluidic resistive pulse sensing (MRPS). The total EV numbers were normalized to total EV‐releasing cells. Means ± SD, *n* = 3 independent experiments, paired student's *t*‐test. (f) Western blot analysis of CD63, CD9 and CD81 from lysates of con‐EVs and TGF‐β‐EVs for the comparison of EV amount with different normalization ways (equal cell number, equal particle number and equal protein amount). Representative of *n* = 3 experiments. (g) Quantification of relative protein expression from the equal number of EV‐releasing cells of western blot results (f). Means ± SD, *n* = 3 independent experiments, unpaired student's *t*‐test. (h) Multivesicular body‐plasma membrane (MVB‐PM) fusion activity (events/minute) in the vehicle and TGF‐β‐treated cells measured by pHluorin‐CD63 reporter assay. Means ± SD, unpaired student's *t*‐test. Representative of *n* = 3 experiments. (i) Size distribution of con‐EVs and TGF‐β‐EVs characterized by cryogenic electron microscopy (cryo‐EM). Medians with interquartile ranges are shown in the graph. Unpaired student's *t*‐test. (j) Representative cryo‐EM images of non‐dense EVs and dense EVs from con‐EVs and TGF‐β‐EVs. The scale bar represents 50 nm. (k) Representative cryo‐EM images of bi‐layered EVs and multi‐layered EVs from con‐EVs and TGF‐β‐EVs. The scale bar represents 50 nm. (l) Quantification of non‐dense EVs and dense EVs in con‐EVs and TGF‐β‐EVs from *n* = 422 EVs for both conditions. (m) Quantification of bi‐layered EVs and multi‐layered in con‐EVs and TGF‐β‐EVs from *n* = 422 EVs for both conditions. **p < 0.01, ***p < 0.001.

To explore the effect of TGF‐β on EV release, we used MDA‐MB‐231 breast cancer cells, a frequently used experimental model in TGF‐β and EV‐related cancer biology research (Tkach et al., 2022). We confirmed their responsiveness to TGF‐β as analysed by SMAD2 phosphorylation levels (Persson et al., 1998) (Figure S1a) and SMAD3‐driven transcriptional (CAGA)_12_‐EGFP (Marvin et al., 2022) reporter activity (Figure S1b) upon TGF‐β challenge. Next, we pretreated these cells with TGF‐β or vehicle control for 1 day in complete medium and subsequently changed to the serum‐free medium supplemented with TGF‐β or vehicle control and incubated for another 2 days for subsequent EV isolation. The EVs were isolated from conditioned medium by ultrafiltration and SEC (Figure 1a). We first used the commonly used method of NTA to estimate the concentrations of control cells derived‐EVs (con‐EVs) and TGF‐β‐treated cells derived‐EVs (TGF‐β‐EVs). We normalized the total EV numbers to the total live cell numbers to compare the EV release in two conditions of cells. Upon treatment with TGF‐β, the MDA‐MB‐231 cells released less EVs than the vehicle control treated cells (Figure 1b,c). To investigate whether this trend also applies to smaller EVs below NTA's limit of detection (LOD), we used MRPS to measure the EV concentrations and particle size distributions. As measured by MRPS with a 70‐nm LOD, the normalized EV concentration was also decreased in TGF‐β‐EVs, consistent with the NTA results (Figure 1d,e).

The tetraspanins CD63, CD81, and CD9 are frequently used markers of EVs (Kalluri & McAndrews, 2023). Although their expression levels vary among different subtypes of EVs (Mathieu et al., 2021), we used their expression of the whole population of EVs to estimate EV numbers. Different normalization criteria were applied to compare the expression of CD63, CD81, and CD9. Specifically, when EV proteins were loaded on the polyacrylamide gel based on the equal number of EV‐secreting cells, a reduction of the expression of all three tetraspanins was observed upon TGF‐β treatment, indicating fewer EVs were released from TGF‐β‐treated cells (Figure 1f,g). When the equal protein amount of EVs was loaded, the expression of tetraspanins was also lower in TGF‐β‐EVs, indicating that there are less EVs in TGF‐β‐EVs, and TGF‐β‐EVs are loaded with higher amount of protein (Figure 1f). When an equal number of EVs were loaded, the CD63 was also decreased in TGF‐β‐EVs. This could be caused by the change in the expression of protein cargo in EVs, the changing ratio of EV subpopulations (less exosomes in an equal number of EVs in TGF‐β‐EVs), or the missing data of smaller EVs due to LOD (Figure 1f).

To further investigate whether TGF‐β inhibited the secretion of exosomes, we used a pHluorin‐CD63 reporter assay (Ma et al., 2018). This assay enables the detection of a single MVB‐PM fusion event, and thereby the release of exosomes, by detecting the resulting fluorescence signal using high‐speed TIRF microscopy. For the analysis of EV secretion especially for exosomes, this reporter can eliminate the biases caused by the potential variability in EV recovery during isolation and the limitations of EV detection techniques (Ma et al., 2018). We found that TGF‐β inhibited the fusion activity of MVB‐PM, which explains how TGF‐β reduces the secretion of exosomes (Figure 1h; Figure S1c and Videos S1 and S2).

The EV concentrations in the conditioned medium depend not only on the EV secretion ability of cells but are also influenced by their re‐uptake. Therefore, we tested whether TGF‐β can affect cellular EV uptake. Ideally, equal particle concentration of con‐EVs and TGF‐β‐EVs should be labelled and an equal number of labelled EVs should be given to the cells for the comparison of EV uptake. However, it is difficult to make this ideal comparison due to the LOD of current techniques including MRPS. Thus, we decided to provide only the PKH67‐labelled EVs from non‐treated MDA‐MB‐231 cells to the control and TGF‐β‐pre‐treated MDA‐MB‐231 cells. A dye control was also included to exclude the possibility of the presence and the uptake of dye aggregates. No significant difference in EV uptake was observed between control and TGF‐β‐treated cells (Figure S1d).

To explore whether TGF‐β affects the size distribution and morphology of EVs, which are potentially associated with their composition and biological functions (Van De Wakker et al., 2024), we performed cryogenic electron microscopy (cryo‐EM) for con‐EVs and TGF‐β‐EVs derived from MDA‐MB‐231 cells. Notably, we found that upon TGF‐β stimulation, the median particle diameter decreased (Figure 1i; Figure S1e), implying the composition and functions may also change in TGF‐β‐EVs. We further classified EVs into dense (full of dense materials) or non‐dense (partial dense materials or translucent content) (Figure 1j). Around two‐fold increase in dense EVs was observed in TGF‐β‐EVs compared to con‐EVs. The latter suggests that TGF‐β may play a role in cargo sorting into EVs, including lipids and proteins (Figure 1l). Next, the size distribution of each EV subtype was compared. The median particle diameters of non‐dense and dense EVs in the TGF‐β‐EV group were smaller than the corresponding subtypes in con‐EVs (Figure S1f). This latter finding was consistent with the trend from the whole EV population (Figure 1i). In addition, the median particle diameter of non‐dense EVs was lower than that of dense EVs in both con‐EVs and TGF‐β‐EVs (Figure S1f), implying that the dense materials in EVs may correlate with the size of EVs. We also classified EVs based on the number of lipid bilayers, that is, one or multiple bilayers (Figure 1k). Interestingly, we found TGF‐β decreased the percentage of multi‐layered EVs compared to con‐EVs (Figure 1m). Taken together, our results show that TGF‐β can inhibit EV release and affect EV morphology.

### 3.2 TGF‐β inhibits the EV release in breast cancer cells by downregulating RAB27B expression

**FIGURE 2:**
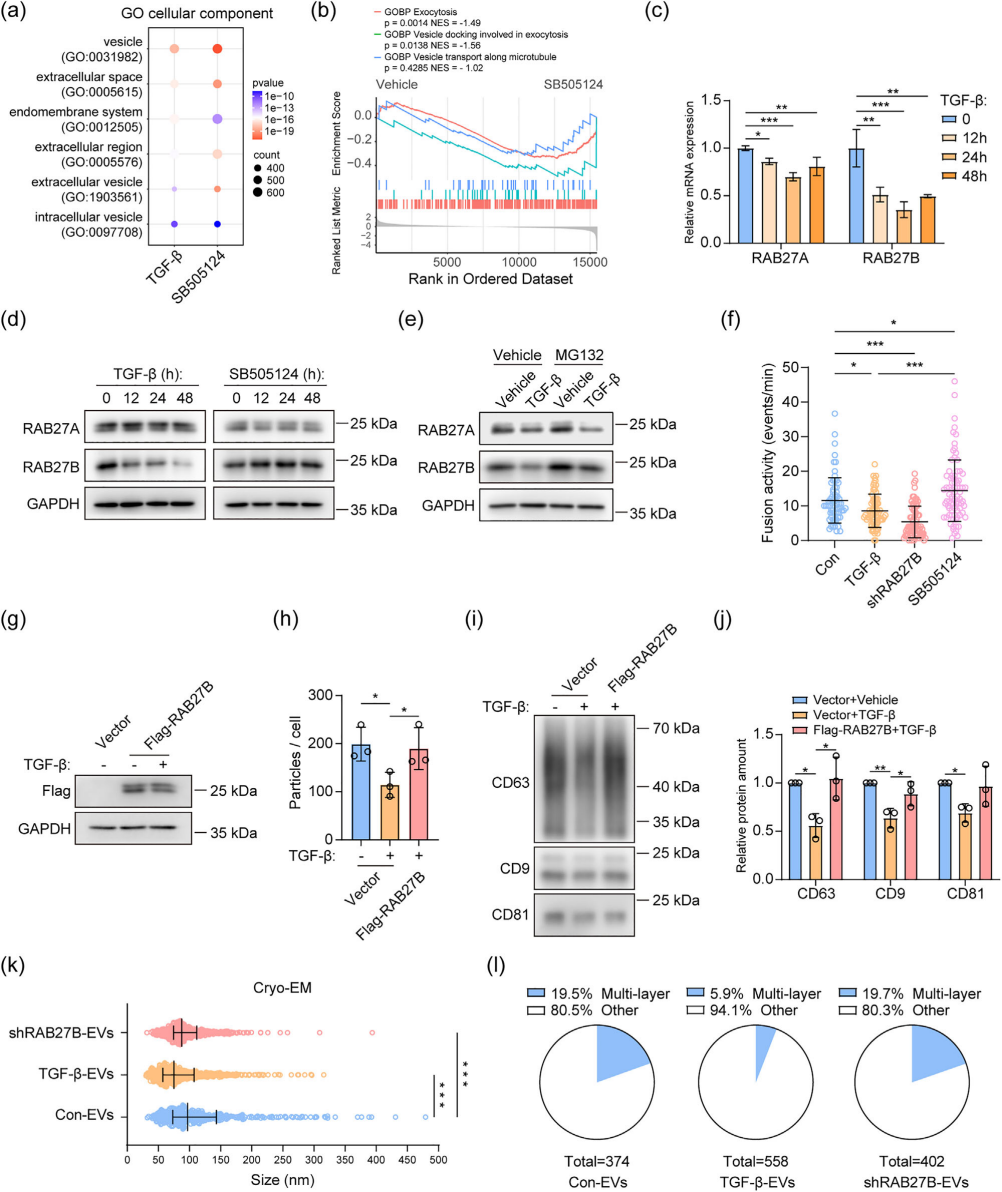
TGF‐β affects the EV release and morphology mediated by downregulating RAB27B expression. (a) Gene Ontology (GO) enrichment analysis of the RNA‐seq data with comparing TGF‐β 24 hours vs. 0 hour and SB505124 24 h vs. 0 h from MDA‐MB‐231 cells. Fisher's exact test with false discovery rate (FDR) correction. (b) Gene set enrichment analysis (GSEA) of EV release‐related pathways with the comparison of SB505124 0 h vs. 24 h. (c) Real‐time PCR analysis of the mRNA expression of *RAB27A* and *RAB27B* in response to TGF‐β at 0, 12, 24, and 48 h. Means ± SD, *n* = 3 biological replicates, one‐way ANOVA with Dunnett's test. Representative of *n* = 3 experiments. (d) RAB27A and RAB27B protein expression in response to TGF‐β and SB505124 (1 µM) at 0, 12, 24, and 48 h. Representative of *n* = 3 experiments. (e) Protein expression of RAB27A and RAB27B in response to TGF‐β with or without MG132 (5 µM) treatment. Representative of *n* = 3 experiments. (f) pHluorin‐CD63 reporter analysis of the MVB‐PM fusion activity in vehicle, TGF‐β, SB505124 (1 µM) treated cells and RAB27B‐depleted cells. Means ± SD, one‐way ANOVA with Tukey's test. Representative of *n* = 3 experiments. (g) Western blot analysis of the expression of Flag‐RAB27B in response to TGF‐β. Representative of *n* = 3 experiments. (h) EV release of control and Flag‐RAB27B overexpressed cells with or without TGF‐β treatment. The total EV numbers were normalized to total EV‐releasing cells. Means ± SD, *n* = 3 independent experiments, one‐way repeated measures ANOVA with Tukey's test. (i) Western blot analysis of CD63, CD9 and CD81 expression from EV lysates based on the equal cell number for the comparison of EV amount. Representative of *n* = 3 experiments. (j) Quantification of relative protein expression of western blot results (i). Means ± SD, *n* = 3 independent experiments, one‐way ANOVA with Tukey's test. (k) Size distribution of con‐EVs, TGF‐β‐EVs and shRAB27B‐EVs characterized by cryo‐EM. Medians with interquartile ranges are shown in the graph. One‐way ANOVA with Tukey's test. (l) Quantification of multi‐layered EVs and other types of EVs in con‐EVs, TGF‐β‐EVs and shRAB27B‐EVs. *p < 0.05, **p < 0.01, ***p < 0.001.

To further explore the mechanism underlying the decreased EV release by TGF‐β stimulation, we analysed our previous RNA‐seq transcriptional profiling data on MDA‐MB‐231 cells (Fan et al., 2023). By subjecting the data to gene ontology (GO) analysis, we found that some EV‐related pathways were significantly affected when the MDA‐MB‐231 cells were treated with TGF‐β or selective small molecule TβRI kinase inhibitor SB505124 for 24 h (Figure 2a; Appendix S1). As the SB505124 compound treatment response contributed to more significant changes in EV‐related pathways, the gene set enrichment analysis (GSEA) was performed to investigate whether inhibiting TGF‐β signalling pathway with SB505124 affects specific EV release‐related pathways. The exocytosis and vesicle docking involved in exocytosis pathways were significantly upregulated by SB505124 treatment, and the vesicle transport along the microtubule pathway was also upregulated but not significantly (Figure 2b). By looking into the mRNA expression of specific genes related to these pathways, we found that the mRNAs of *RAB27A* and *RAB27B* were downregulated by TGF‐β and upregulated by SB505124 treatment. Both RAB27 proteins have key roles in regulating the trafficking and docking process of MVBs (Ostrowski et al., 2010). The inhibitory effect by TGF‐β on mRNA expression levels of *RAB27A* and *RAB27B* was further validated by real‐time PCR, and they both decreased significantly when MDA‐MB‐231 cells were treated with TGF‐β for 12, 24, and 48 h, with a higher mRNA reduction for *RAB27B* (Figure 2c). Next, the protein expression of RAB27A and RAB27B was analysed in response to TGF‐β and SB505124 treatment. The protein expression of RAB27B kept decreasing from 12 to 48 h in the presence of TGF‐β, and conversely increased with SB505124 treatment. However, the protein level of RAB27A remained very stable (Figure 2d), even though we prolonged the TGF‐β treatment for up to 8 days (Figure S2a). We assume that stable RAB27A expression may be achieved by the reduced protein degradation, to maintain the normal biological processes in cells (Rothman, 2010). Therefore, we used MG132 to inhibit the proteasome‐mediated protein degradation, and the reduced RAB27A protein expression was observed in the presence of TGF‐β as well as RAB27B, indicating that the synthesis of RAB27A was indeed inhibited by TGF‐β (Figure 2e). As the protein level of RAB27A remained stable in response to TGF‐β, we focused on the role of RAB27B in further functional studies and whether TGF‐β inhibits EV release by downregulating RAB27B expression. We generated two shRNA‐mediated *RAB27B* knockdown cell lines and validated the expression of RAB27B (Figure S2b). Next, pHluorin‐CD63 reporter assay was performed to compare the effect of TGF‐β, SB505124, and RAB27B depletion on the secretion of exosomes. The MVB‐PM fusion activity was suppressed by TGF‐β and RAB27B depletion, and RAB27B depletion showed a stronger effect, which was consistent with the protein level of RAB27B from TGF‐β‐treated cells and RAB27B‐depleted cells (Figure 2f; Figure S2b). In contrast, the SB505124 increased the MVB‐PM fusion activity (Figure 2f).

Next, we constructed the RAB27B overexpression cell line. Notably, the protein expression of Flag epitope tagged‐RAB27B with the heterologous CMV promoter was not affected by TGF‐β, indicating that TGF‐β regulates the transcription of RAB27B, but not its protein stability (Figure 2g). Furthermore, the overexpression of RAB27B rescued the inhibitory effect of TGF‐β on EV release, by restoring the normalized particle concentration of EVs and the expression of tetraspanins (Figure 2h–j).

We next examined whether TGF‐β can downregulate the expression of RAB27B in other breast cancer cell lines including BT‐549, MCF‐7, MDA‐MB‐436, SUM149, BT‐474, and cervical cancer cells HeLa. We confirmed their responsiveness to TGF‐β by analysing SMAD2 phosphorylation levels following TGF‐β treatment (Figure S2c). Only in BT‐549 cells the protein expression of RAB27B decreased significantly (Figure S2d). Accordingly, the EV release in BT‐549 cells was reduced by TGF‐β examined by NTA assay and the expression of tetraspanins normalized to equal EV‐secreting cells (Figure S2e,f). In addition, the protein levels of RAB27B remained relatively stable in other cell lines (Figure S2d). EV release in other cell lines was also compared by normalized EV concentration. Consistent with the lack of effect on RAB27B expression, there was almost no difference between control and TGF‐β‐treated cells (Figure S2e).

To explore whether the loss of RAB27B affects the uptake of EVs, which can also affect the EV concentration in conditioned medium, we added PKH67‐labelled EVs from non‐treated MDA‐MB‐231 cells to the control cells, TGF‐β‐treated cells, and RAB27B‐depleted cells, also with a group of cells added with PKH67 dye control. Similar to previous results shown in Figure S1d, the TGF‐β treatment and RAB27B depletion did not significantly differ in EV uptake (Figure S2g).

As we found TGF‐β can decrease the median particle size of EVs before (Figure 1i), we examined whether this effect was mediated by the loss of RAB27B. We found that the median particle size of shRNA‐mediated *RAB27B* knockdown cell‐derived EVs (shRAB27B‐EVs), as determined by cryo‐EM, was significantly decreased compared to the control. This latter effect was slightly higher for TGF‐β‐EVs (Figure 2k), implying that the TGF‐β‐induced inhibition was partially mediated by RAB27B downregulation. Although we still observed that TGF‐β reduced the ratio of multi‐layered EVs, the ratio of multi‐layered EVs in shRAB27B‐EVs remained almost unchanged compared to con‐EVs, suggesting that the effect of TGF‐β on the release of multi‐layered EVs was independent of RAB27B (Figure 2l). Collectively, these data show that TGF‐β downregulates the expression of RAB27B in a cell type‐dependent manner, and thereby suppresses EV release.

### 3.3 TGF‐β suppresses the transcription of *RAB27B* mediated by SMAD3

**FIGURE 3:**
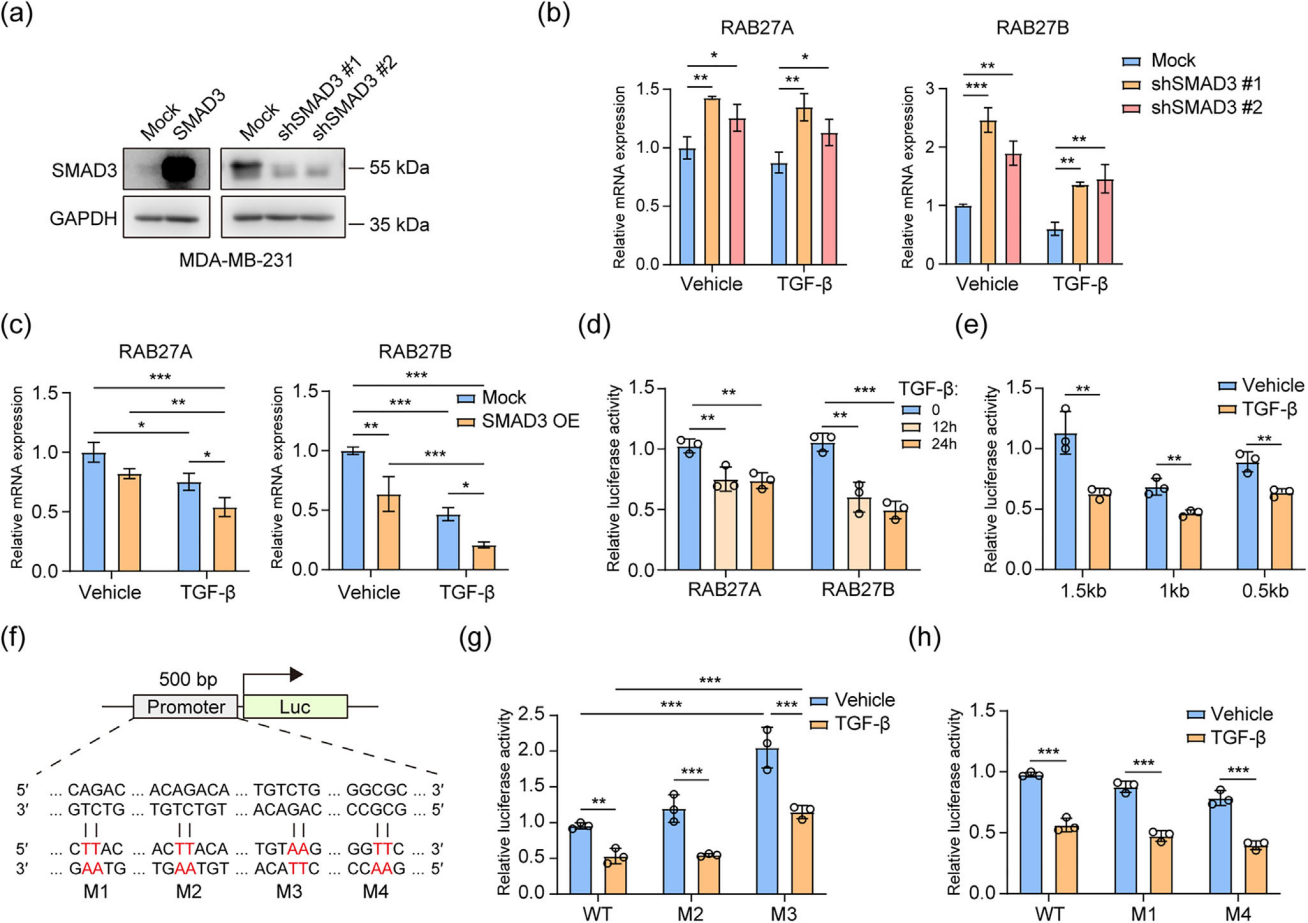
TGF‐β regulates the transcription of *RAB27B* in a SMAD3‐dependent mechanism. (a) Validation of SMAD3 expression in SMAD3 overexpression and shRNA‐mediated knockdown cells by western blot. (b) Real‐time PCR analysis of the mRNA expression of *RAB27A* and *RAB27B* in WT and *SMAD3* knockdown cells with vehicle or TGF‐β treatment. Means ± SD, *n* = 3 biological replicates, one‐way ANOVA with Dunnett's test. Representative of *n* = 3 experiments. (c) Real‐time PCR analysis of the mRNA expression of *RAB27A* and *RAB27B* in mock and SMAD3 overexpressed cells with vehicle or TGF‐β treatment. Means ± SD, *n* = 3 biological replicates, two‐way ANOVA with Tukey's test. Representative of *n* = 3 experiments. (d) The effect of TGF‐β on transcriptional activity of 1500 bp *RAB27A* and *RAB27B* promoter regions detected by luciferase reporter assay. Means ± SD, *n* = 3 biological replicates, one‐way ANOVA with Dunnett's test. Representative of *n* = 3 experiments. (e) The effect of TGF‐β on transcriptional activity of truncated *RAB27A* and *RAB27B* promoter regions detected by luciferase reporter assay. Means ± SD, *n* = 3 biological replicates, unpaired student's *t*‐test. Representative of *n* = 3 experiments. (f) Schematic of the luciferase reporter constructs containing 500 bp *RAB27B* promoter with mutations. (g) The effect of TGF‐β on transcriptional activity of *RAB27B* promoter regions with M2 or M3 mutation detected by luciferase reporter assay. Means ± SD, *n* = 3 biological replicates, two‐way ANOVA with Tukey's test. Representative of *n* = 3 experiments. (h) The effect of TGF‐β on transcriptional activity of *RAB27B* promoter regions with M1 or M4 mutation detected by luciferase reporter assay. Means ± SD, *n* = 3 biological replicates, unpaired student's *t*‐test. Representative of *n* = 3 experiments. *p < 0.05, **p < 0.01, ***p < 0.001.

Once TGF‐β binds to the TGF‐β receptors and phosphorylates TβRI, both SMAD and non‐SMAD signalling pathways can be activated (Colak & Ten Dijke, 2017). To explore by which pathway TGF‐β regulates the expression of RAB27A and RAB27B, we generated *SMAD3* shRNA‐mediated knockdown and SMAD3 overexpression cell lines and validated the depletion and ectopic expression of SMAD3 (Figure 3a). Upon *SMAD3* knockdown, the mRNA expression levels of both *RAB27A* and *RAB27B* were significantly increased, and TGF‐β stimulation was still capable of suppressing the mRNA expression of *RAB27A* and *RAB27B*, especially for *RAB27B* (Figure 3b). By contrast, the overexpression of SMAD3 mimicked the effect of TGF‐β stimulation. It suppressed the mRNA expression of *RAB27A* and *RAB27B*, and the TGF‐β stimulation can further strengthen the effect of SMAD3 overexpression (Figure 3c). Taken together, the TGF‐β regulates the expression of RAB27A and RAB27B, at least in part, in a SMAD3‐dependent manner.

To investigate whether SMAD3 regulates the mRNA expression of *RAB27A* and *RAB27B* by controlling the transcription of these two genes, we constructed luciferase transcriptional reporters containing 1500 bp promoter regions upstream of the transcriptional start sites of *RAB27A* and *RAB27B*. The luciferase activities of both genes were suppressed by TGF‐β, with an increasing inhibitory effect after a longer treatment time (Figure 3d). There was a higher inhibitory effect on the promoter of *RAB27B* compared to *RAB27A*; these results are consistent with the mRNA expression levels regulated by TGF‐β (Figure 2c). Therefore, we further explored the possible sites where SMAD3 regulates the transcription of *RAB27B*. First, 1000 and 500 bp truncations of the *RAB27B* promoter were tested for luciferase reporter assay, and both decreased in response to TGF‐β treatment (Figure 3e). Next, we mutated the potential SMAD3 DNA binding sites within the 500 bp promoter region, including three CAGA/CAGA‐like motifs and one 5‐bp GC motif (Itoh et al., 2019; Martin‐Malpartida et al., 2017) (Figure 3f). As M2 and M3 sites are more conserved CAGA motifs, we tested these two sites first with the reporters encoding the single mutation of each site. The reporter with the M3 mutation significantly increased the luciferase activity compared to the WT promoter in both control and TGF‐β treatment conditions (Figure 3g). However, the M1, M2, and M4 did not affect the transcriptional activity of the *RAB27B* promoter, indicating that SMAD3 suppresses the transcription of *RAB27B* on M3 site (Figure 3g,h). Although the mutation on M3 site can upregulate the overall transcriptional activity, TGF‐β still showed an inhibitory effect, suggesting that TGF‐β/SMAD3 may also regulate the transcription of *RAB27B* by other indirect mechanisms.

### 3.4 TGF‐β and RAB27B affect the protein cargo sorting in MVBs

**FIGURE 4:**
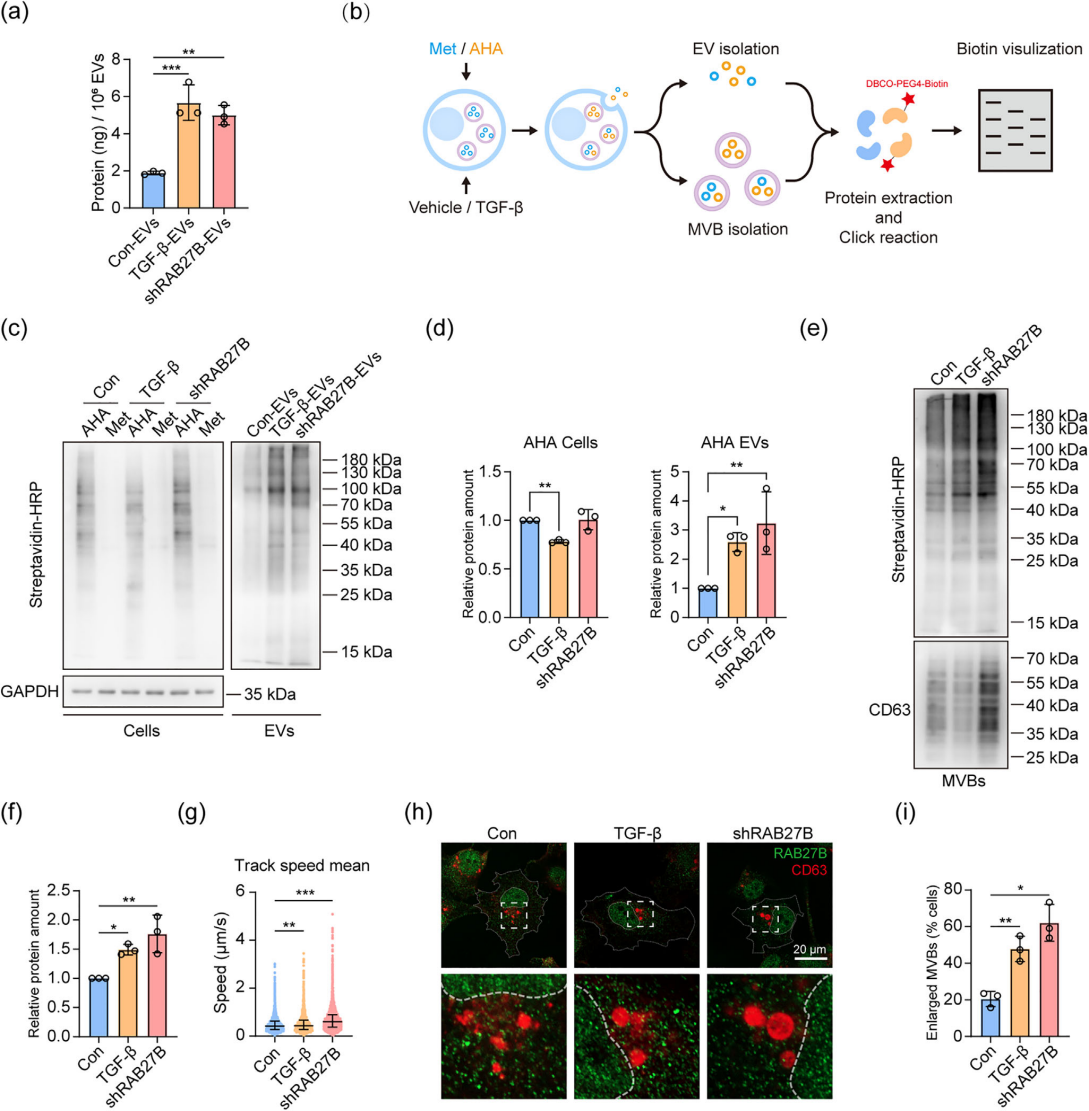
TGF‐β regulates the sorting of protein cargo in EVs mediated by downregulating RAB27B expression. (a) Protein quantification of con‐EVs, TGF‐β‐EVs, and shRAB27B‐EVs normalized to equal number of EVs. Means ± SD, *n* = 3 biological replicates, one‐way ANOVA with Dunnett's test. Representative of *n* = 3 experiments. (b) Schematic of utilizing click chemistry to detect the newly synthesized proteins in EVs and MVBs. (c) Western blot analysis of the newly synthesized protein in vehicle, TGF‐β‐treated cells and *RAB27B* knockdown cells, and con‐EVs, TGF‐β‐EVs and shRAB27B‐EVs. The lysates from the equal protein amount of cells and equal number of EVs were loaded on gel for western blot assay. Representative of *n* = 3 experiments. (d) Quantification of relative protein expression of the western blot results (c). Means ± SD, *n* = 3 independent experiments, one‐way ANOVA with Dunnett's test. (e) Western blot analysis of the newly synthesized protein in MVBs from the vehicle, TGF‐β‐treated cells and *RAB27B* knockdown cells. The lysates from equal number of cells were loaded on gel for western blot assay. Representative of *n* = 3 experiments. (f) Quantification of relative protein expression of the western blot results (e). Means ± SD, *n* = 3 independent experiments, one‐way ANOVA with Dunnett's test. (g) Track speed of mScarlet‐CD63 in vehicle, TGF‐β‐treated cells and *RAB27B* knockdown cells measured by high‐speed total internal reflection fluorescence (TIRF) microscopy. Medians with interquartile ranges, one‐way ANOVA with Dunnett's test. Representative of *n* = 3 experiments. (h, i) Representative super‐resolution microscopy images of CD63 (red) and RAB27B (green) in the vehicle, TGF‐β‐treated cells and *RAB27B* knockdown cells. The regions containing MVBs were magnified. The scale bar represents 20 µm. The percentages of cells containing enlarged MVBs were quantified. Means ± SD, *n* = 3 independent experiments, one‐way repeated measures ANOVA with Dunnett's test. *p < 0.05, **p < 0.01, ***p < 0.001.

In addition to investigating EV release, we also examined whether the protein content of EVs can be affected by TGF‐β stimulation and RAB27B depletion, as the protein composition of EVs is directly associated with their function. We normalized the amount of EV protein to the particle numbers of EVs (as measured by MRPS), and we found that TGF‐β and the loss of RAB27B significantly increased the protein amount in EVs derived from MDA‐MB‐231 and BT‐549 cells (Figure 4a; Figure S3a). To further explore whether RAB27B regulates the protein sorting into EVs, we used click chemistry to label the newly synthesized proteins in cells with L‐azidohomoalanine (AHA) (Figure 4b). The labelling efficiency of AHA was examined using DBCO‐Alexa 488 fluorescent dye at different time points, and a higher fluorescence signal was observed after incubating cells with AHA for 8 h (Figure S3b). Next, EVs were isolated and EV proteins were extracted and detected using equal EV numbers. We found that TGF‐β and *RAB27B* knockdown both upregulated the amount of nascent protein in EVs (Figure 4c,d). In cell lysates, the amount of newly synthesized protein was not affected by RAB27B depletion, but it was slightly decreased by TGF‐β treatment (Figure 4c,d).

Since TGF‐β and the depletion of RAB27B can suppress the MVB‐PM fusion events and subsequently exosome secretion (Figure 2f), we examined whether TGF‐β and RAB27B also affect the protein cargo in exosomes. If TGF‐β and RAB27B regulate the sorting of protein cargo in exosomes, the protein content should be changed in MVBs as exosomes originate from MVBs. To investigate this, we isolated the MVBs and validated their purity by analysing the levels of different organelle markers (Figure S3c,d). Subsequently, we used click chemistry to track the newly synthesized proteins in MVBs (Figure 4b). We observed that the total protein amount of MVBs was increased by TGF‐β and *RAB27B* knockdown (Figure S3e). Moreover, the newly synthesized protein in MVBs was elevated by TGF‐β and *RAB27B* knockdown (Figure 4e,f). Of note, the CD63 levels in MVBs from *RAB27B* knockdown cells was increased, whereas TGF‐β decreased the CD63 levels slightly (Figure 4e). We then analysed CD63 levels in total cell lysates, and the trend was consistent with the CD63 levels from MVBs (Figure S3f).

RAB27B plays an important role in MVB trafficking, and the loss of RAB27B in HeLa cells was shown to increase the moving speed of MVBs along microtubules, which potentially decreases the occurrence of MVB docking (Ostrowski et al., 2010). We found that TGF‐β can also increase the velocity of MVB movements. *RAB27B* knockdown showed higher significance, which is in line with the lower RAB27B expression in *RAB27B* knockdown cells (Figure 4g; Videos S3–S5), compared to TGF‐β‐mediated RAB27B downregulation. Furthermore, we examined whether TGF‐β and *RAB27B* knockdown affect the subcellular localization and morphology of MVBs. Whereas the localization of MVBs remained similar with TGF‐β stimulation or *RAB27B* knockdown, TGF‐β and *RAB27B* knockdown enlarged the size of MVBs and increased the proportion of cells with the enlarged MVBs (Figure 4h,i). In addition, TGF‐β and *RAB27B* knockdown did not affect the subcellular localization and morphology of early endosomes and lysosomes (Figure S3g). Together, TGF‐β and the loss of RAB27B can increase the protein amount in exosomes and affect the morphology and trafficking of MVBs.

### 3.5 TGF‐β changes the protein composition in EVs mediated by RAB27B

**FIGURE 5:**
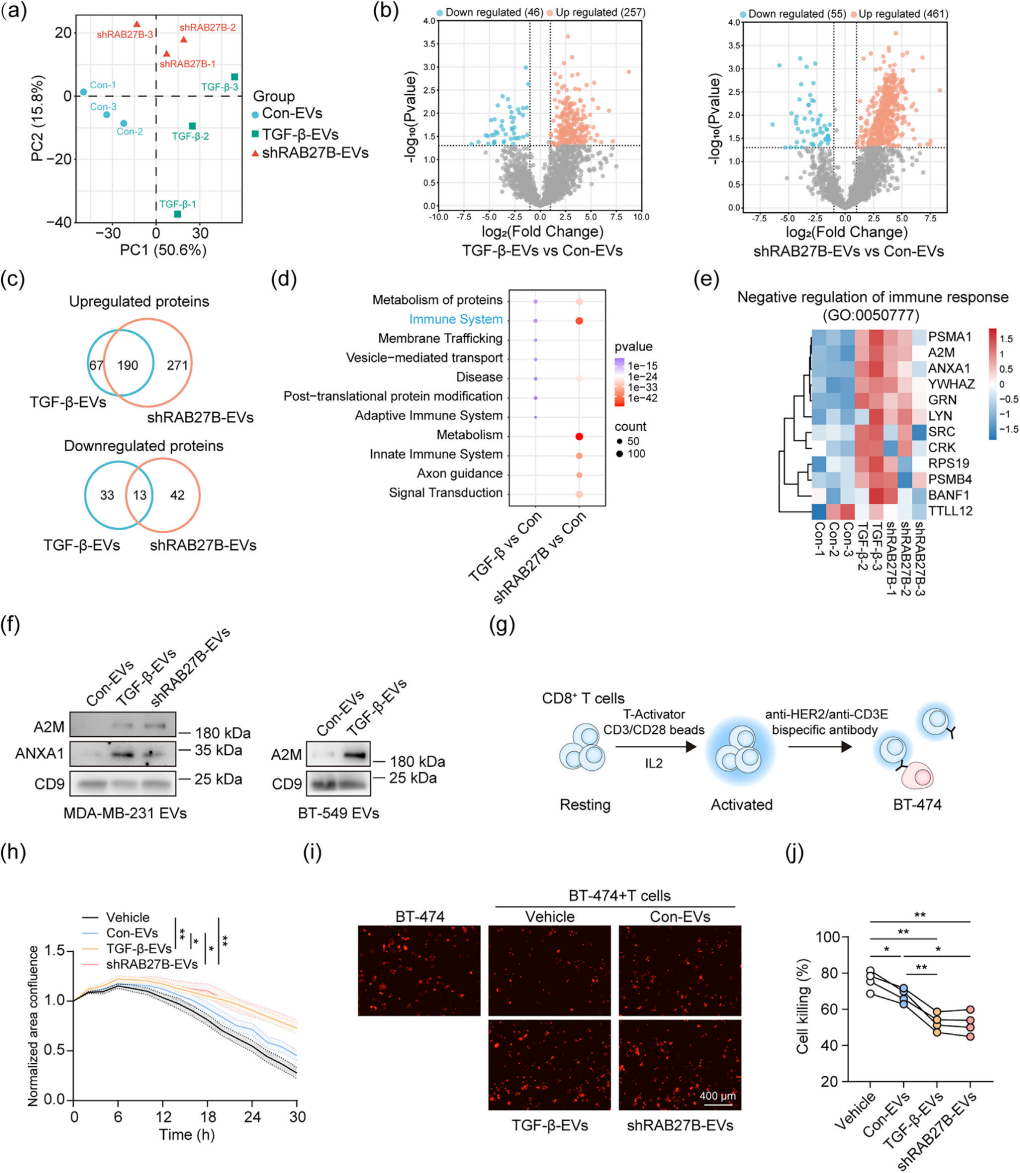
TGF‐β and RAB27B affect the protein composition and functions of EVs. (a) Principal component analysis (PCA) of proteins identified in con‐EVs, TGF‐β‐EVs and shRAB27B‐EVs. (b) Volcano plots showing differential protein expression comparing TGF‐β‐EVs vs. con‐EVs and shRAB27B‐EVs vs. con‐EVs. (c) Venn diagram showing the number of upregulated and downregulated proteins in TGF‐β‐EVs and shRAB27B‐EVs comparing to con‐EVs. (d) Reactome pathway analysis of the upregulated proteins in TGF‐β‐EVs and shRAB27B‐EVs comparing to con‐EVs. Fisher's exact test with false discovery rate (FDR) correction. The presented pathways are from the top 10 enriched pathways. (e) Heatmap analysis of the expression of the proteins related to negative regulation of immune response (GO: 0050777) in EV samples. The expression values of proteins are *z*‐score transformed. (f) Western blot analysis of the expression of A2M and ANXA1 in EVs based on the equal EV number. (g) Schematic description of CD8^+^ T cells and BT‐474 co‐culture system mediated by anti‐HER2/anti‐CD3E bispecific antibody. (h, i) Real‐time recording of the growth of BT‐474 cells (red) co‐cultured with CD8^+^ T cells, and representative images of different conditions were shown. The BT‐474 cells were co‐cultured with CD8^+^ T cells and EVs or vehicle control for 30 h. The scale bar represents 400 µm. One‐way ANOVA with Tukey's test. Representative of *n* = 4 donors. (j) Quantification of the cytotoxicity of CD8^+^ T cells in a co‐culture system. The percentages of cancer cells killed by CD8^+^ T cells were calculated using the cancer cell monoculture as controls. One‐way repeated measures ANOVA with Tukey's test with *n* = 4 donors. *p < 0.05, **p < 0.01.

To further validate if TGF‐β regulates the sorting of specific cargo in EVs mediated by downregulating RAB27B expression, we performed label‐free quantitative proteomic analysis based on an equal number of EVs measured by MRPS. Proteomic contents of the biological replicates of each condition clustered together and separated from EVs of other conditions (Figure 5a). Next, the differentially expressed proteins (DEPs) in TGF‐β‐EVs and shRAB27B‐EVs were compared to con‐EVs. As expected, more upregulated proteins were detected in TGF‐β‐EVs and shRAB27B‐EVs than downregulated proteins (Figure 5b). Notably, 190 identical proteins were upregulated in TGF‐β‐EVs and shRAB27B‐EVs, which account for 74% of total upregulated proteins in TGF‐β‐EVs and 41% of total upregulated proteins in shRAB27B‐EVs, suggesting that TGF‐β regulates the sorting of protein cargo in EVs largely mediated by downregulating RAB27B expression (Figure 5c). For the downregulated proteins, there were 13 proteins shared by TGF‐β‐EVs and shRAB27B‐EVs (Figure 5c). Of note, TGF‐β3, the recombinant TGF‐β ligand that we used for cell treatments, was not present in the detected EV proteins.

Next, we investigated the biological functions related to the upregulated proteins in TGF‐β‐EVs and shRAB27B‐EVs. Notably, the immune system pathway was significantly enriched in both comparisons, ranking at the top among all enriched pathways, which suggested an important role of TGF‐β‐EVs and shRAB27B‐EVs in immune regulation (Figure 5d; Appendix S2). Moreover, proteins related to the negative regulation of immune response were upregulated in TGF‐β‐EVs and shRAB27B‐EVs (Figure 5e). We selected A2M and ANXA1 to further validate their protein levels in EVs, as the upregulation of A2M and ANXA1 is related to immune suppression (Araújo et al., 2021; Vandooren & Itoh, 2021). An upregulation of protein levels of A2M and ANXA1 was observed in TGF‐β‐EVs and shRAB27B‐EVs compared to con‐EVs derived from MDA‐MB‐231 cells (Figure 5f). In EVs derived from BT‐549 cells, A2M was also upregulated in TGF‐β‐EVs, whereas the level of ANXA1 was too low to be detected (Figure 5f). Taken together, these data suggest that TGF‐β‐EVs and shRAB27B‐EVs may have suppressive effect on immune response.

### 3.6 TGF‐β‐EVs and shRAB27B‐EVs share similar immunosuppressive effects

CD8^+^ T cells are major effectors in the anticancer immune response (Waldman et al., 2020), and emerging studies have shown the immunosuppressive effect of tumour cell‐derived EVs (Marar et al., 2021). Based on the proteomic results, we investigated whether TGF‐β‐EVs and shRAB27B‐EVs can suppress the functions of CD8^+^ T cells, by treating CD8^+^ T cells with an equal number of EVs (4 × 10^8^ particles/mL) from different conditions. First, the effect of EVs on the cancer cell‐killing ability of activated T cells was examined using a co‐culture system with BT‐474 breast cancer cells and CD8^+^ T cells mediated by anti‐HER2/anti‐CD3E bispecific antibody (Figure 5g). By tracking the growth of BT‐474 cells in real‐time, we found that more cancer cells survived during co‐culture in EV treatment groups, with higher significance in TGF‐β‐EV and shRAB27B‐EV treatment groups (Figure 5h,i). TGF‐β‐EVs and shRAB27B‐EVs significantly suppressed the breast cancer‐killing ability of T cells compared to vehicle control and con‐EVs, implying that the EV cargo altered by TGF‐β and RAB27B depletion gave EVs a more immunosuppressive effect (Figure 5j). In addition, we confirmed that EVs from MDA‐MB‐231 cells did not affect the proliferation of BT‐474, indicating that TGF‐β‐EVs and shRAB27B‐EVs increased cell number by impairing the cancer‐killing ability of T cells in co‐culture (Figure S4a).

Next, the production of inflammatory cytokines IFN‐γ and TNF‐α by activated CD8^+^ T cells following EV treatments was tested. TGF‐β‐EVs and shRAB27B‐EVs significantly inhibited the production of IFN‐γ and TNF‐α, whereas con‐EVs had no significant effect (Figure S4b–d). Moreover, the amount of secreted IFN‐γ and TNF‐α was reduced by TGF‐β‐EVs and shRAB27B‐EVs (Figure S4e,f).

In addition, we examined the uptake of fluorescence labelled EVs in T cells using flow cytometry, and the populations of T cells with increased fluorescence signal were detected in EV treatment groups, and there was no significant difference in EV uptake between EV treatment groups (Figure S4g,h). We further confirmed that PKH67‐labelled EVs were internalized in CD8^+^ T cells using microscopy (Figure S4i). Taken together, TGF‐β‐EVs and shRAB27B‐EVs more potently impaired the function of CD8^+^ T cells compared to con‐EVs, particularly their cancer cell‐killing ability.

## 4 DISCUSSION

TGF‐β is a key regulator in tumour development, and emerging studies have shown the interplay between TGF‐β and the biological functions of EVs in the tumour microenvironment. This study systematically analysed how TGF‐β influences the release and morphology of EVs from breast cancer cells. Our findings revealed that TGF‐β downregulates the release of EVs by inhibiting the transcription of *RAB27B* mediated by SMAD3. TGF‐β and the loss of RAB27B promote higher quantity of protein loading with EVs, especially exosomes, which inhibit CD8^+^ T cell‐mediated killing of breast cancer cells.

EVs are highly heterogeneous due to their size distributions and origins. Here, we used several techniques to characterize the EV release, including NTA, MRPS, and EV marker levels as detected by western blot and pHluorin‐CD63 reporter assay. Each technique has its advantages and shortcomings (Bebelman et al., 2020; Welsh et al., 2024). To our best knowledge, all the current techniques are unable to accurately measure the absolute concentrations of EVs in their broadest size range, partly due to constraints in the LOD or throughput of the techniques. So far, NTA is still the most widely used technique for EV quantification. By using NTA, we observed decreased EV concentrations normalized to equal EV‐secreting cells in the TGF‐β‐EV group. To include more smaller EVs in the measurement, we used MRPS to characterize EVs, which can more accurately measure small EVs, and the results were consistent with what we observed from NTA.

Comparing the protein levels of EV tetraspanin markers is another way to estimate the EV secretion. CD63, CD81, and CD9 are the most commonly used markers for EV characterization and have been used as indicators of EV release (Ostrowski et al., 2010). We observed lower expression of these markers normalized to the equal number of parental cells. Besides detecting CD63, CD81, and CD9 using antibodies directly, other approaches have been developed in the last decade to detect the EV release by engineering CD63, such as GFP‐CD63, NanoLuc‐CD63, and ThermoLuc‐CD63, which can be independent of EV isolation and enable the high‐throughput drug or gene screening affecting the EV secretion (Adem et al., 2021; Bebelman et al., 2023; Datta et al., 2018; Gupta et al., 2020). Another technique to directly detect EV secretion based on CD63 is pHluorin‐CD63, which reacts with a sudden increase in fluorescence intensity when pH shifts from acidic to neutral and can reflect the secretion of exosomes. Using this method, we found that the secretion of exosomes was inhibited by TGF‐β treatment. This finding motivated us to investigate the biogenesis and secretion of exosomes affected by TGF‐β, and we do not exclude the possibility that TGF‐β influences the release of plasma membrane‐derived EVs in this study.

The biogenesis of exosomes is a multi‐step and highly regulated process with many proteins involved (Arya et al., 2024). The GTPases RAB27A and RAB27B are known to regulate MVB trafficking and exosome release in different steps (Ostrowski et al., 2010). We found that TGF‐β/SMAD3 signalling can inhibit the transcription of both genes, especially for *RAB27B* in MDA‐MB‐231 cells. We further demonstrated the importance of SMAD3 binding sites (at least in part) in mediating the TGF‐β‐induced repression of *RAB27B* promoter activity. Of note, the effect of TGF‐β on the inhibition of RAB27A and RAB27B expression is cell type‐dependent.

Although the release of EVs was inhibited by TGF‐β and RAB27B depletion, the quantity of protein cargo was highly upregulated in EVs derived from MDA‐MB‐231 cells. By applying click chemistry, we observed an increased amount of newly synthesized protein cargo in EVs. To understand whether exosomes were specifically affected by RAB27B, we isolated the MVBs and observed a higher amount of nascent proteins in them from TGF‐β‐treated cells and *RAB27B* knockdown cells, indicating that RAB27B indeed plays a role in the sorting of protein cargo in MVBs. However, we do not exclude the possibility that the cargo in ectosomes can also be affected by RAB27B. Next, we performed quantitative proteomics based on the equal EV number measured by MRPS and observed more upregulated than downregulated proteins. Notably, a large proportion of upregulated proteins in TGF‐β‐EVs was shared by shRAB27B‐EVs, indicating that TGF‐β affects the sorting of protein cargo largely mediated by RAB27B.

Interestingly, a recent study indicated that the loss of RAB27B inhibited EV release and altered the protein composition of EVs from leukaemia stem cells (Chen et al., 2024). In addition, we found that the upregulated quantity of protein in MVBs may correlate with the enlarged size of MVBs in TGF‐β‐treated cells and RAB27B‐depleted cells. The enlarged size of MVBs was also observed upon RAB27B depletion in leukaemia stem cells, with unchanged subcellular localization (Chen et al., 2024). However, in HeLa cells, RAB27A (but not RAB27B) silencing induced an enlargement of MVBs, whereas RAB27B affected the subcellular localization of MVBs (Ostrowski et al., 2010). These findings further indicate that the function of RAB27B could be cell type‐dependent. During MVB maturation, the trans‐Golgi network (TGN) supplies endosomal sorting complexes required for transport (ESCRT) for cargo sorting during ILV biogenesis and other selective cargo from Golgi to MVBs (Arya et al., 2024; Kwon et al., 2016). The small GTPases play an important role in this TGN‐endosome trafficking, including RAB11 and RAB27B (Savina et al., 2002). Therefore, TGF‐β stimulation and RAB27B depletion may change the protein composition in MVBs and exosomes by affecting TGN‐endosome trafficking.

The recomposed EV cargo induced by TGF‐β stimulation and RAB27B depletion leads to increased immunosuppressive effects in CD8^+^ T cells. The cancer cell‐killing capacity and cytokine production of activated CD8^+^ T cells were suppressed by TGF‐β‐EVs and shRAB27B‐EVs. We suppose this inhibition effect was caused by a bulk of cargo in EVs, rather than one single protein, as multiple upregulated proteins in TGF‐β‐EVs and shRAB27B‐EVs were correlated with immunosuppression. Besides, TGF‐β‐EVs and shRAB27B‐EVs may also exert immunosuppressive effects on other immune cell types in the tumour microenvironment.

We characterized the morphology of EVs from different conditions by cryo‐EM. The TGF‐β‐EVs and shRAB27B‐EVs are generally smaller than con‐EVs. The size of ILVs, the precursors of EVs, can be influenced by the ILV cargo and its formation mechanism, including ESCRT‐dependent and independent processes (Arya et al., 2024; Willms et al., 2016). In line with the proteomics results, we speculate that TGF‐β stimulation and RAB27B depletion caused the formation of different types of ILVs, but whether their size difference is a result of their cargo or of other mechanisms controlling their formation remains to be investigated. In addition, we found that the proportion of multi‐layered EVs was reduced by TGF‐β, but not by RAB27B depletion, implying that TGF‐β can regulate the formation of multi‐layered EVs in a RAB27B‐independent manner. Until now, still very little is known about the biogenesis and biological functions of multi‐layered EVs (Broad et al., 2023); our research paves the way to further investigate the formation of multi‐layered EVs in a TGF‐β‐related manner.

The lipid composition of the EV membrane is highly heterogeneous in different EV subpopulations and origins, which mainly consists of cholesterol, phosphatidylcholine, sphingolipids, and so forth (Ghadami & Dellinger, 2023). TGF‐β is a key factor promoting epithelial‐mesenchymal transition (EMT) and reprogramming the lipid metabolism including membrane lipids during EMT (Din et al., 2024; Soukupova et al., 2021). Notably, cholesterol, phosphatidylcholine, and sphingolipids have all been reported to promote EMT by different mechanisms (Cumin et al., 2021; Jiang et al., 2019; Seok et al., 2021). In our study, we found TGF‐β reduced the EV release and size but upregulated the amount of protein cargo in EVs to suppress the T cell functions. We speculate this is a sophisticated mechanism for cancer cells to save membrane lipids from EVs to promote cell metastasis during EMT, and these reprogrammed EVs are loaded with more immunosuppressive cargo to promote cancer development.

Overall, our findings revealed that TGF‐β signalling inhibits EV release but promotes the loading of protein cargo in EVs mediated by downregulating RAB27B expression, and thereby induces immunosuppression in CD8^+^ T cells.

## AUTHOR CONTRIBUTIONS

**Chao Li**: Data curation (equal); formal analysis (equal); investigation (lead); methodology (equal); project administration (equal); software (equal); validation (equal); visualization (equal); writing—original draft (lead). **Agustin Enciso‐Martinez**: Formal analysis (supporting); investigation (equal); methodology (equal); visualization (equal); writing—review and editing (equal). **Roman I. Koning**: Data curation (supporting); formal analysis (supporting); investigation (equal); methodology (equal); software (equal); writing—review and editing (supporting). **Mona Shahsavari**: Formal analysis (supporting); investigation (supporting); methodology (supporting); writing—review and editing (supporting). **Peter ten Dijke**: Funding acquisition (equal); investigation (equal); project administration (equal); supervision (lead); writing—review and editing (lead).

## CONFLICT OF INTEREST STATEMENT

The authors declare no competing interests.

## Supporting information



Supporting Information

Supporting Information

Supporting Information

Video S1

Video S2

Video S3

Video S4

Video S5

## Data Availability

All relevant data are available from the authors. The mass spectrometry proteomics data have been deposited to the ProteomeXchange Consortium via the PRIDE partner repository with the dataset identifier PXD052799.
