# Supplementary material for: TGF‐β regulates the release of breast cancer cell‐derived extracellular vesicles and the sorting of their protein cargo by downregulating RAB27B expression
Source: J Extracell Vesicles. 2024 Dec 26;13(12):e70026. doi: 10.1002/jev2.70026 (PMC11669950; doi:10.1002/jev2.70026)
Supplement: Supplementary file 1 — Supporting Information [file JEV2-13-e70026-s008.docx]

Supplementary Information

**TGF-β regulates the release of breast cancer cell-derived extracellular vesicles and the sorting of their protein cargo by downregulating RAB27B expression**

Chao Li^1^, Agustin Enciso Martinez^1,2^, Roman I. Koning^3^, Mona Shahsavari^2,4,5,6,7^ & Peter ten Dijke^1*^

^1^ Oncode Institute and Department of Cell and Chemical Biology, Leiden University Medical Center, Leiden, Netherlands.

^2^ Amsterdam Vesicle Center, Biomedical Engineering and Physics, Amsterdam University Medical Center, Netherlands

^3^ Electron Microscopy Facility, Department of Cell and Chemical Biology, Leiden University Medical Center, Leiden, Netherlands.

^4^ Laboratory of Experimental Clinical Chemistry, Amsterdam UMC, University of Amsterdam, Amsterdam, Netherlands.

^5^ Biomedical Engineering and Physics, Amsterdam UMC, University of Amsterdam, Amsterdam, Netherlands.

^6^ Amsterdam Cardiovascular Sciences, Atherosclerosis and Ischemic Syndromes, Amsterdam, Netherlands.

^7^ Cancer Center Amsterdam, Imaging and Biomarkers, Amsterdam, Netherlands.

*Correspondence to Peter ten Dijke ([p.ten_dijke@lumc.nl](mailto:p.ten_dijke@lumc.nl))


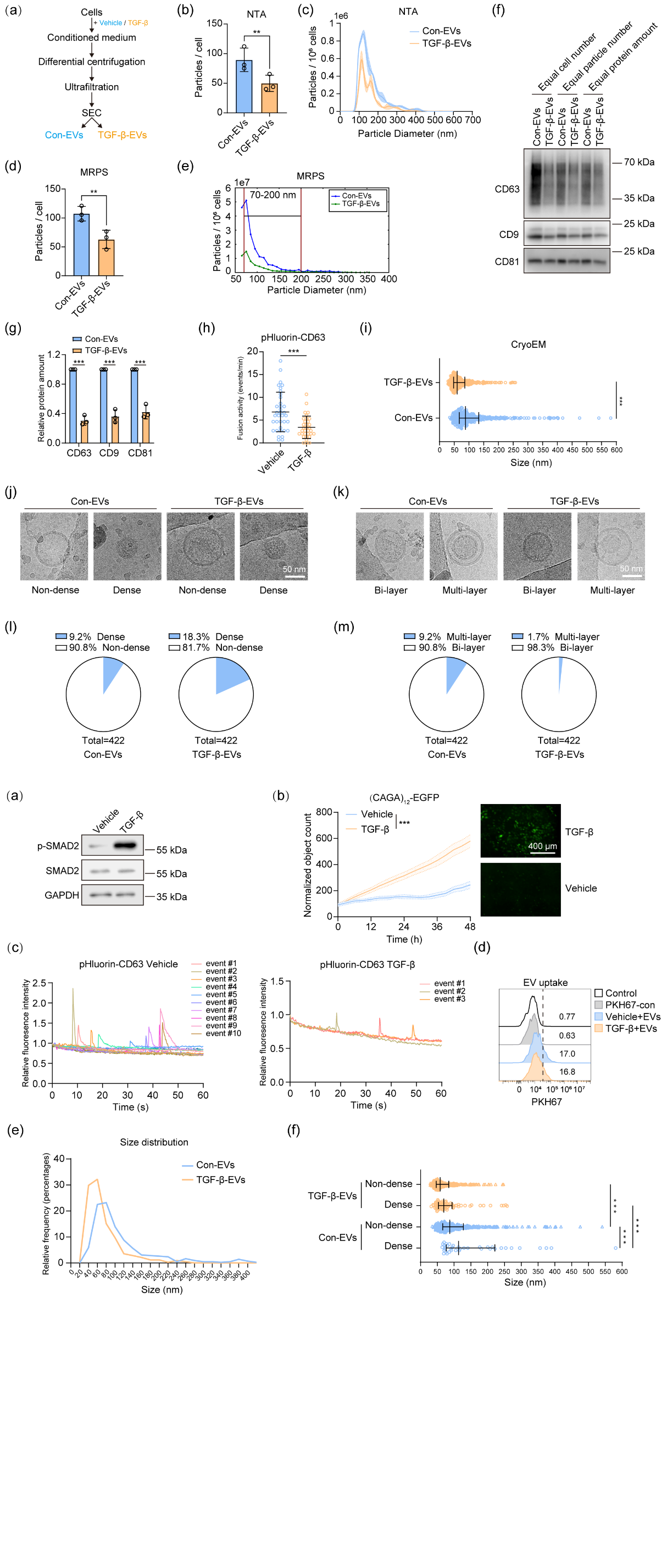


**Supplementary Fig. 1 TGF-β affects the EV release and morphology.**

(a) Western blot analysis of p-SMAD2 expression in response to TGF-β in MDA-MB-231 cells. Cells were treated with TGF-β or vehicle buffer for 2 hours. Representative of n = 3 experiments. (b) Real-time imaging of (CAGA)_12_-SMAD3 transcriptional EGFP reporter transfected cells with vehicle and TGF-β treatments. The quantification of normalized green object counts is shown on the left. Means ± SD, student’s *t*-test. Representative images are shown on the right. The scale bar represents 400 μm. Representative of n = 3 experiments. (c) Representative graphs of fluorescence intensity of the spots where the MVB-PM fusion occurred in one cell from the vehicle and TGF-β treatment groups. (d) Quantifying the uptake of PKH67-labeled EVs in vehicle/TGF-β-treated cells measured by flow cytometry. The values represent the percentages of cells internalized with PKH67-labeled EVs. Representative of n = 3 experiments. (e) Size distribution of relative frequency of con-EVs and TGF-β-EVs characterized by cryo-EM. (f) Size distribution of non-dense EVs and dense EVs in con-EVs and TGF-β-EVs characterized by cryo-EM. Medians with interquartile ranges are shown in the graph. Two-way ANOVA with Tukey’s test. ****p* < 0.001.


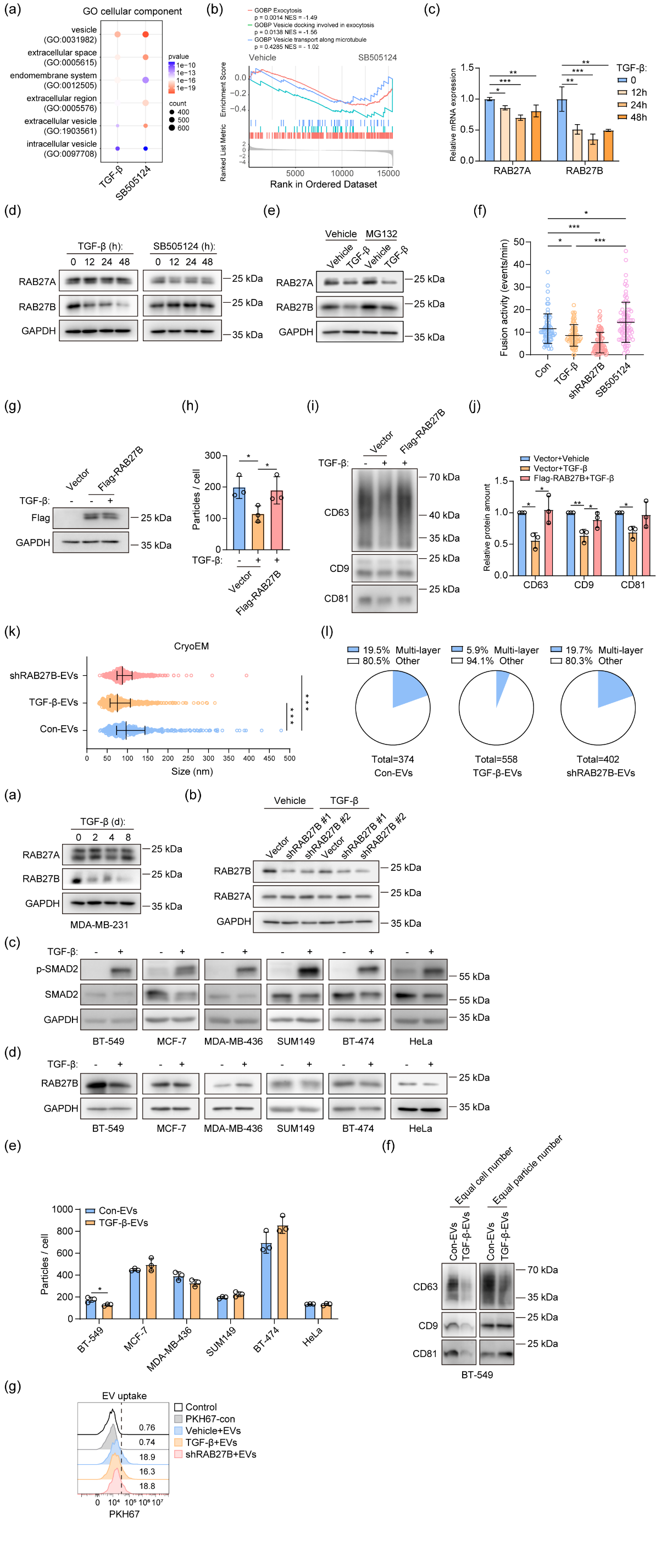


**Supplementary Fig. 2 TGF-β affects the EV release and morphology mediated by downregulating RAB27B expression.**

(a) RAB27A and RAB27B protein expression in response to TGF-β for 0, 12, 24 and 48 h. Representative of n = 3 experiments. (b) Protein expression of RAB27B and RAB27A in WT and *RAB27B* knockdown cells with or without TGF-β treatment. Representative of n = 3 experiments. (c) Protein expression of p-SMAD2 in response to TGF-β in BT-549, MCF-7, MDA-MB-436, SUM149, BT-474 and HeLa cells. Cells were treated with TGF-β or vehicle buffer for 2 hours. Representative of n = 3 experiments. (d) Protein expression of RAB27B in BT-549, MCF-7, MDA-MB-436, SUM149, BT-474 and HeLa cells with or without TGF-β treatment. Representative of n = 3 experiments. (e) EV release of BT-549, MCF-7, MDA-MB-436, SUM149, BT-474 and HeLa cells with or without TGF-β treatment characterized by nanoparticle tracking analysis (NTA). The total EV numbers were normalized to total EV-releasing cells. Means ± SD, n = 3 technical replicates, unpaired student’s *t*-test. Representative of n = 2 experiments. (f) Western blot analysis of CD63, CD9 and CD81 expression from lysates of con-EVs and TGF-β-EVs derived from BT-549 cells based on the equal cell number and equal particle number for the comparison of EV amount. Representative of n = 3 experiments. (g) Quantifying the uptake of PKH67-labeled EVs in the vehicle, TGF-β-treated cells and RAB27B-depleted cells measured by flow cytometry. The values represent the percentages of cells internalized with PKH67-labeled EVs. Representative of n = 3 experiments. **p* < 0.05.


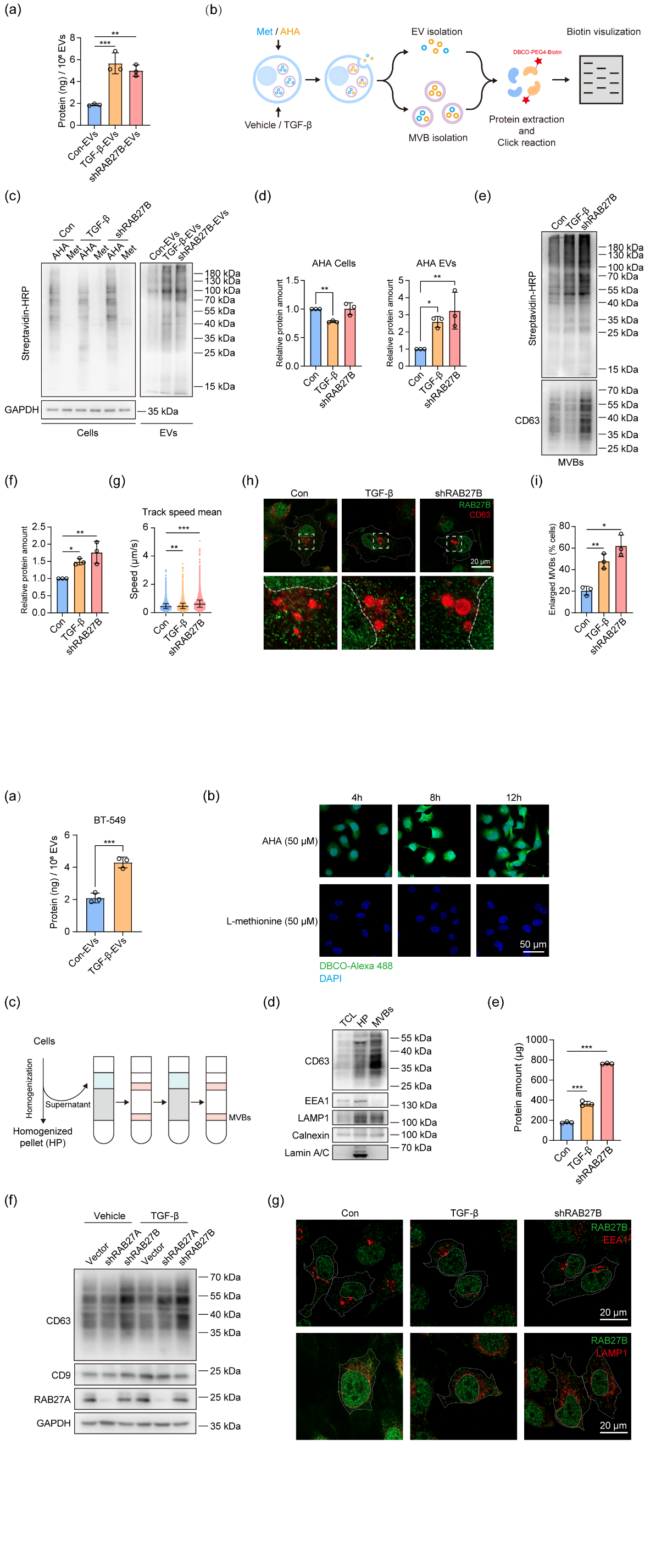


**Supplementary Fig. 3 TGF-β regulates the sorting of protein cargo in EVs mediated by downregulating RAB27B expression**

(a) Protein quantification of con-EVs and TGF-β-EVs from BT-549 cells. The amount of EV protein was normalized to EV numbers. Means ± SD, n = 3 biological replicates, one-way ANOVA with Dunnett’s test. Representative of n = 3 experiments. (b) Representative confocal microscopy images of the detection of newly synthesized proteins in MDA-MB-231 cells. Cells were treated with AHA for 4h, 8h and 12h and newly synthesized proteins were detected with DBCO-Alexa 488. The scale bar represents 50 μm. (c) Schematic of the MVB isolation workflow. (d) Validation of MVB isolation by western blot analysis comparing the levels of different organelle makers from total cell lysates (TCL), homogenized pellets (HPs) and MVBs. Representative of n = 3 experiments. (e) Protein quantification of MVBs isolated from equal number of the vehicle, TGF-β-treated and *RAB27B* knockdown cells. Means ± SD, n = 3 biological replicates, one-way ANOVA with Dunnett’s test. Representative of n = 3 experiments. (f) Western blot analysis of CD63 of *RAB27A* and *RAB27B* knockdown cells with or without TGF-β treatment. Representative of n = 3 experiments. (g) Representative super-resolution microscopy images of EEA1 (red), LAMP1 (red) and RAB27B (green) in vehicle, TGF-β-treated cells and *RAB27B* knockdown cells. The scale bar represents 20 μm. Representative of n = 3 experiments. ****p* < 0.001.


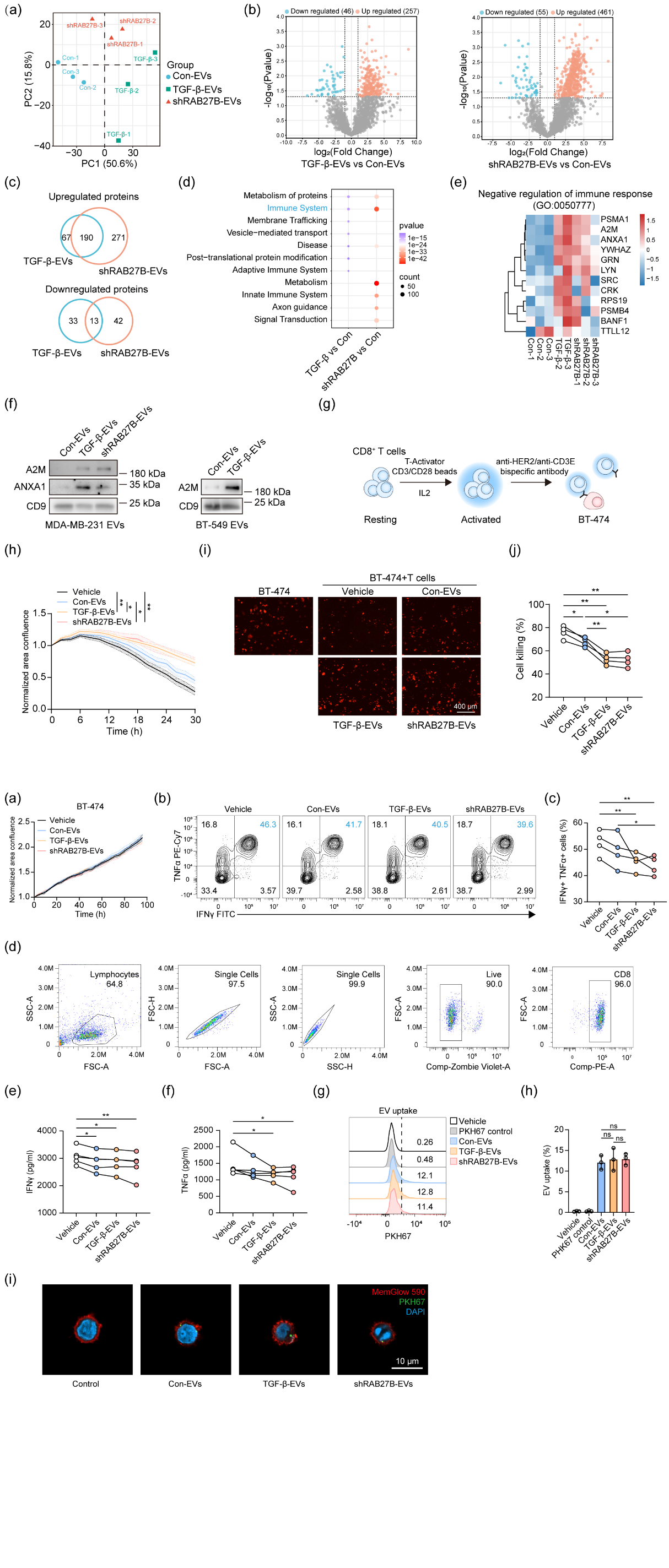


**Supplementary Fig. 4 TGF-β-EVs and shRAB27B-EVs suppress T cell functions.**

(a) Proliferation of BT-474 cells recorded by Incucyte for 4 days of treatment. Means ± SD, n = 3 biological replicates, one-way ANOVA with Dunnett’s test. Representative of n = 3 experiments. (b, c) Quantifying IFN-γ and TNF-α production as measured by intracellular cytokine staining, and representative flow cytometry plots are shown. T cells were treated with EVs or vehicle control for 2 days. One-way repeated measures ANOVA with Tukey’s test with n = 4 donors. (d) Gating strategy to identify lymphocyte single live CD8^+^ T cells used in Supplementary Fig.4b and c. (e, f) Secreted IFN-γ and TNF-α measured by ELISA assay. T cells were treated with EVs or vehicle control for 2 days. One-way repeated measures ANOVA with Tukey’s test with n = 5 donors. (g, h) Representative flow cytometry analysis of the uptake of PKH67-labeled EVs in activated CD8^+^ T cells. The percentages of CD8^+^ T cells containing PKH67-labeled EVs were quantified. One-way repeated measures ANOVA with Tukey’s test with n = 3 donors. (i) Confocal microscopy imaging of the uptake of PKH67-labeled EVs in activated CD8^+^ T cells. The cell membranes were labeled with MemGlow 590. Representative of n = 2 experiments. The scale bar represents 10 μm. **p* < 0.05, ***p* < 0.01.

**Supplementary Table 1. The list of antibodies for western blot**

| Antibody | Clone | Company | Cat # | Target species | Dilution |
| --- | --- | --- | --- | --- | --- |
| CD63 | MX-49.129.5 | Santa Cruz | Sc-5275 | Human | 1:1000 |
| CD9 | HI9a | Biolegend | 312102 | Human | 1:1000 |
| CD81 | B-11 | Santa Cruz | Sc-166029 | Human | 1:1000 |
| RAB27A | D7Z9Q | Cell signaling | 69295S | Human | 1:1000 |
| RAB27B | Polyclonal | Cell signaling | 44813S | Human | 1:1000 |
| GAPDH | 6C5 | Millipore | MAB374 | Human | 1:2000 |
| SMAD3 | EP568Y | Epitomics | 1735-1 | Human | 1:1000 |
| EEA1 | 1G11 | Thermofisher | 14-9114-80 | Human | 1:1000 |
| LAMP1 | H4A3 | BD Bioscience | 555798 | Human | 1:1000 |
| Calnexin | Polyclonal | Abcam | Ab22595 | Human | 1:1000 |
| Lamin A/C | 636 | Vector Laboratories | VP-L550 | Human | 1:1000 |
| Phospho-SMAD2 (Ser465/467) | 138D4 | Cell signaling | 3108 | Human | 1:1000 |
| SMAD2 | EP784Y | Epitomics | 1736-1 | Human | 1:1000 |
| Flag | M2 | Sigma | F1804 |  | 1:1000 |
| Streptavidin-HRP |  | Cell signaling | 3999s |  | 1:2000 |

**Supplementary Table 2. The list of primers used in this study**

| Primer: | Target species | Sequence (5′ - 3′): |
| --- | --- | --- |
| GAPDH-F | Human | TGCACCACCAACTGCTTAGC |
| GAPDH-R | Human | GGCATGGACTGTGGTCATGAG |
| RAB27A-F | Human | ACAACAGTGGGCATTGATTTCA |
| RAB27A-R | Human | AAGCTACGAAACCTCTCCTGC |
| RAB27B-F | Human | TAGACTTTCGGGAAAAACGTGTG |
| RAB27B-R | Human | AGAAGCTCTGTTGACTGGTGA |

**Supplementary Table 3. The list of antibodies for flow cytometry**

| Antibody | Fluorochrome | Clone | Company | Cat # | Target species | Dilution |
| --- | --- | --- | --- | --- | --- | --- |
| IFN-γ | FITC | 4S.B3 | Biolegend | 502506 | Human | 1:30 |
| TNF-α | PE-Cyanine 7 | MAb11 | Thermofisher | 25-7349-82 | Human | 1:65 |
| CD8a | PE | RPA-T8 | Thermofisher | 12-0088-42 | Human | 1:30 |
